# Hybridization promotes growth performance by altering rumen microbiota and metabolites in sheep

**DOI:** 10.3389/fvets.2024.1455029

**Published:** 2024-09-25

**Authors:** Rui Zhang, Liwa Zhang, Xuejiao An, Jianye Li, Chune Niu, Jinxia Zhang, Zhiguang Geng, Tao Xu, Bohui Yang, Zhenfei Xu, Yaojing Yue

**Affiliations:** ^1^Lanzhou Institute of Husbandry and Pharmaceutical Sciences, Chinese Academy of Agricultural Sciences, Lanzhou, China; ^2^Sheep Breeding Engineering Technology Research Center of Chinese Academy of Agricultural Sciences, Lanzhou, China; ^3^Key Laboratory of Animal Genetics and Breeding on Tibetan Plateau, Ministry of Agriculture and Rural Affairs, Lanzhou, China; ^4^Qingyang Research Institute of Agricultural Sciences, Qingyang, China; ^5^Agricultural and Rural Comprehensive Service Center of Gengwan Township, Qingyang, China

**Keywords:** hybridization, rumen, metagenome, growth performance, sheep

## Abstract

Hybridization can substantially improve growth performance. This study used metagenomics and metabolome sequencing to examine whether the rumen microbiota and its metabolites contributed to this phenomenon. We selected 48 approximately 3 month-old male ♂Hu × ♀Hu (HH, *n* = 16), ♂Poll Dorset × ♀Hu (DH, *n* = 16), and ♂Southdown × ♀Hu (SH, *n* = 16) lambs having similar body weight. The sheep were fed individually under the same nutritional and management conditions for 95 days. After completion of the trial, seven sheep close to the average weight per group were slaughtered to collect rumen tissue and content samples to measure rumen epithelial parameters, fermentation patterns, microbiota, and metabolite profiles. The final body weight (FBW), average daily gain (ADG), and dry matter intake (DMI) values in the DH and SH groups were significantly higher and the feed-to-gain ratio (F/G) significantly lower than the value in the HH group; additionally, the papilla height in the DH group was higher than that in the HH group. Acetate, propionate, and total volatile fatty acid (VFA) concentrations in the DH group were higher than those in the HH group, whereas NH_3_-N concentration decreased in the DH and SH groups. Metagenomic analysis revealed that several *Prevotella* and *Fibrobacter* species were significantly more abundant in the DH group, contributing to an increased ability to degrade dietary cellulose and enrich their functions in enzymes involved in carbohydrate breakdown. *Bacteroidaceae bacterium* was higher in the SH group, indicating a greater ability to digest dietary fiber. Metabolomic analysis revealed that the concentrations of rumen metabolites (mainly lysophosphatidylethanolamines [LPEs]) were higher in the DH group, and microbiome-related metabolite analysis indicated that *Treponema bryantii* and *Fibrobacter succinogenes* were positively correlated with the LPEs. Moreover, we found methionine sulfoxide and N-methyl-4-aminobutyric acid were characteristic metabolites in the DH and SH groups, respectively, and are related to oxidative stress, indicating that the environmental adaptability of crossbred sheep needs to be further improved. These findings substantially deepen the general understanding of how hybridization promotes growth performance from the perspective of rumen microbiota, this is vital for the cultivation of new species and the formulation of precision nutrition strategies for sheep.

## 1 Introduction

Hybridization is an important method of breeding sheep that can produce heterosis, resulting in a new breed that is stronger or performs better than its parents (1). Binary hybridization in the cultivation of new varieties of mutton sheep has been frequently reported in China (2). Even though three-way crossbreeding is commonly used in pig production and for developing new breeds (3, 4), relevant research is still in the early stages of selecting suitable species on mutton sheep industry (5). Compared with the former, the latter could effectively utilize the heterosis of three hybrid individuals, producing a significantly stronger effect. Hu is a native sheep breed with perennial estrus, high fecundity, high lactation, good parenting traits, and strong adaptability; therefore, they are often used as crossbreeding ewes for mutton sheep, however, their meat production rate and dressing percentage are unsatisfactory (6, 7). Poll Dorset is native to Australia and New Zealand, has the characteristics of fast growth, early maturity, and perennial estrus, and is considered the main parent for hybridization in the Chinese sheep breeding industry (8). Southdown is considered the best breed for meat quality in the United Kingdom due to its ideal meat structure, early maturity, easy breeding, good carcass quality, and dressing percentage > 55% (9, 10). Southdown and Poll Dorset are small sheep species that grow rapidly in the early stages and are suitable for producing high-grade fat lambs, which aligns with the current market demand for mutton in China. Therefore, we attempted to cultivate a new breed of sheep by using Poll Dorset and Southdown as sires, crossing them with Hu sheep, and monitoring the production traits of the F_1_ generation.

Growth performance is the main selection trait for breeding new sheep varieties. The rumen microbiota is associated with animal growth and health (11). The rumen is colonized by bacteria, protozoa, archaea, fungi, and viruses, which degrade complex plant fibers and polysaccharides to produce volatile fatty acids (VFAs), microbial proteins, and vitamins, in turn providing nutrients to meet host requirements for maintenance and growth (12, 13). Although diet plays a major role in shaping the gastrointestinal microbiota, recent evidence has revealed host genetics to be an important factor in determining the gut microbiota composition in cattle (13–15). However, research on the effects of host genetics on the rumen microbiota composition of hybridized sheep is limited.

Therefore, in this study, we hypothesized that rumen microbiota composition and function in sheep are affected by host genetics, affecting growth performance in turn. We assessed the rumen microbiota and metabolite profiles of different hybridized sheep raised in the same farm environment, aiming to elucidate the underlying molecular mechanisms from the perspective of rumen microbiota and thus provide a theoretical and practical basis for improving the growth performance of hybridized sheep.

## 2 Materials and methods

### 2.1 Animals, experimental design, and sample collection

This study was conducted at the Qinghuan Mutton Sheep Breeding Company in Huanxian County, Gansu, China from October 2022 to January 2023. The experimental design, procedures, and methods were approved by the Animal Administration and Ethics Committee of the Lanzhou Institute of Husbandry and Pharmaceutical Science of the Chinese Academy of Agricultural Science under permission number 2022–018 and followed the Chinese standards for the use and care of animals. Forty-eight approximately 3 month-old male ♂Hu × ♀Hu (HH, *n* = 16), ♂Poll Dorset × ♀Hu (DH, *n* = 16), and ♂Southdown × ♀Hu (SH, *n* = 16) lambs were selected and raised in a single pen under the same nutritional and management conditions for 95 days (including a 15 day pre-test period). Hu sheep were chosen from 216 approximately 3 month-old male lambs with an average body weight of 22.98 ± 6.37 kg, and F_1_ generation sheep of similar body weight were selected. Poll Dorset and Southdown sires (aged 31 months) generated from embryo transplantation and Hu sires (aged 24 months) were crossed with Hu ewes (parity one or two) by estrus synchronization and artificial insemination. The offspring (one, in case of twins) were confirmed to stem from different fathers. All sheep were fed the same total mixed ratio composed of oat hay (6.08% DM), *Leymus chinensis* (13.04% DM), corn silage (13.53% DM), corn (32.78% DM), megalac (6.08% DM) and grain mixtures (28.49% DM) twice daily at 08:00 and 15:00 (Supplementary Table S1). During the experimental period, all animals had access to feed and water *ad libitum*. Feed intake was recorded daily based on the feed offered and refusals to calculate the average dry matter intake (DMI). Body weight was recorded every 20 d using a digital livestock scale (TCS Electronic Platform Scale, Rongcheng, China) to calculate the average daily gain (ADG) and feed-to-gain ratio (F/G).

At the end of the experiment, seven sheep close to the average weight per group were selected and fasted for 12 h before harvesting. Harvesting was based on standard commercial procedures, individually restrained, exsanguinated, skinned, and gutted. Rumen tissue samples of approximately 2 cm × 2 cm were carefully separated from the left dorsal sac to fixed in 4% paraformaldehyde solution for hematoxylin–eosin (H&E) staining. Then, two portions of 5 mL rumen content were collected from each animal, transferred into a sterile tube, immediately frozen using liquid nitrogen, and stored at −80°C for DNA and metabolite extraction. Another 15 mL rumen content was sampled and stored in a sterilized container at −20°C for evaluation of fermentation parameters. The rumen contents of all animals were collected from the left dorsal sac as a mixture of liquid and solid components. All samples were collected within 30 min of slaughter.

### 2.2 Rumen epithelial parameters

Rumen epithelial parameters, including papilla height, papilla width, and muscle thickness, were obtained using the H&E staining technique (16). The fixed rumen tissues were dehydrated in alcohol, cleared in xylene, and embedded in paraffin. Then the cooling concretionary samples were sectioned at 5 μm thickness and mounted on glass slides. Paraffin-embedded sections were dewaxed with xylene, passed through a graded ethanol series to remove the xylene, rinsed with distilled water, and stained with H&E. Subsequently, the paraffin was sealed with neutral gum after dehydration and immediately examined. Finally, two digital slides were acquired from each animal using a Slide Viewer device (3D HISTECH Ltd.) at two-fold magnification; then, five papillae (excluding damaged ones) were chosen randomly from each digital slide to measure papilla height using ImageJ software (National Institutes of Health, Bethesda, MD, United States). Within those papillae measured for height, three different regions (apical, middle, and basal) were identified to measure papilla width. Each digital slide was divided into five equal parts to measure muscle thickness.

### 2.3 Rumen fermentation parameters

The pH of the rumen was measured immediately after the sheep were slaughtered using an Ark Technology PHS-10 portable acidity meter (Chengdu, China). The NH_3_-N concentration was determined by colorimetry. Briefly, 2 g of rumen content was accurately weighed and mixed with deionized water at a 1:5 ratio. The mixed solution was shaken for 1 h at 105 r/min and centrifuged at 12,000 rpm at 4°C for 20 min. The supernatant was then passed through a 0.45 μm polymer fiber filter membrane and stored for further analysis. Subsequently, the NH_3_-N concentration was measured using a kit (Biosino Biotechnology Co. Ltd., Beijing, China) according to the manufacturer’s instructions and a microplate reader (DR-200BS; Hiwell-Diatek, Wuxi, China). VFA concentrations were measured as previously described (16). Briefly, rumen contents were centrifuged at 5,400 rpm for 10 min. We subsequently uniformly mixed 1 mL of the resulting supernatant and a 0.2 mL 25% metaphosphate solution containing 2-ethylbutyric acid as an internal standard in a new centrifuge tube. This reaction tube was then immersed in an ice bath (30 min) and centrifuged at 10,000 rpm for another 10 min. The supernatant was passed through a 0.22 μm organic phase filter membrane and stored in 2 mL bottles for subsequent analysis. A gas chromatograph (GC 7890A; Agilent Technologies, Santa Clara, CA, United States) fitted with an AT-FFAP capillary column (50 m × 0.32 mm × 0.25 μm; Agilent Technologies) was used to determine VFA concentrations. The column temperature was maintained at 60°C for 1 min, raised by 5°C/min to 115°C, and increased by 15°C/min to 180°C. Notably, the detector and injector temperatures were 260°C and 250°C, respectively.

### 2.4 Metagenome sequencing and bioinformatics analysis

Total genomic DNA was extracted from rumen content sample using the E.Z.N.A. Soil DNA Kit (Omega Biotek, Norcross, GA, United States). The DNA concentration and purity were determined using TBS-380 mini-fluorometer (Promega, Madison, WI, United States) and NanoDrop 2000 spectrophotometer (Thermo Fisher Scientific, Waltham, MA, United States), respectively. The DNA was fragmented to approximately 400 bp for paired-end library construction using a Covaris M220 (Gene Company Limited, Hong Kong, China). Metagenome libraries sequencing was performed using an Illumina NovaSeq 6,000 platform (Illumina Inc., San Diego, CA, United States).

Quality control of the metagenomic sequence reads was performed using the Fastp software (version 0.20.0) to trim the 3′-end and 5′-end of reads, cut low-quality bases, and remove short reads and “N” records (17). The quality-filtered reads were then aligned to the *Ovis aries* reference genome using BWA v 0.7.1 to filter out the host DNA (18). The remaining reads were assembled using Multiple Megahit (version 1.1.2) (19), and contigs with lengths ≥300 bp were selected as the final assembling result for the prediction of open reading frames (ORF) using MetaGene v 0.3.38 (20). Non-redundant contigs were identified using CD-HIT with 95% sequence identity and 90% coverage (21). Original sequencing reads were mapped to predicted genes to estimate their abundance using SOAPAligner v. 2.21 (22). Subsequently, the non-redundant gene catalog was aligned to the NCBI non-redundant protein sequence database using BLASTP (version 2.2.28+) to obtain taxonomic annotations and species abundances (23). Taxonomic profiles were conducted at domain, phylum, genus and species levels, with relative abundances calculated. The PCoA based on Bray–Curtis dissimilarity matrices at domain level was performed. Bacteria with a relative abundance >0.01% and archaea with a relative abundance >0.001% were analyzed. Finally, a BLAST search (version 2.2.28+) with an optimization criterion cutoff of 1e^−5^ was annotated against the Kyoto Encyclopedia of Genes and Genomes (KEGG) database (24). Carbohydrate-active enzyme (CAZyme) annotation was conducted using hmmscan^1^ against CAZy database version 5.0^2^ with an e-value cutoff of 1e^−5^.

### 2.5 Metabolome sequencing and bioinformatics analysis

Rumen sample metabolite extraction was based on previously published procedures (16). The metabolome of the rumen content was analyzed by ultra-performance liquid chromatography (UPLC; Shim-pack UFLC Shimadzu CBM30A; Shimadzu, Kyoto, Japan) and tandem mass spectrometry (MS/MS, QTRAP^®^ 6,500+, SCIEX, Framingham, MA, United States) (25). After obtaining the liquid chromatography–mass spectrometry data of the samples, the extracted ion chromatographic peaks of all metabolites were integrated using MultiQuant software (Applied Biosystems, Foster City, CA, United States). The chromatographic peaks of the metabolites in different samples were corrected using integration (26). The relative concentrations of rumen metabolites were screened by fold change (FC) (FC ≥ 2 or FC ≤ 0.5) and variable importance in projection (VIP) (VIP ≥ 1) to identify the different metabolites. Identified metabolites were annotated using the KEGG compound database (27).

### 2.6 Statistical analysis

All data were checked for normality and outliers using SPSS version 26.0 (IBM Corp., Armonk, NY, United States) before any statistical analyses were conducted. One-way ANOVA and LSD *post-hoc* test was used to analyze the data of growth performance as well as rumen epithelial and fermentation parameters. Spearman’s correlation test was used for correlation analyses. Statistical significance was set to *p* < 0.05. GraphPad Prism version 8.5 (GraphPad Software, La Jolla, CA, United States) drew the statistical maps.

## 3 Results

### 3.1 Hybridization promoted growth performance in Hu sheep

A summary of the growth performance data is presented in Table 1. The initial body weight (IBM) did not differ significantly among the three groups (*p* = 0.156). The DH and SH groups exhibited greater ADG than did the HH group (*p* = 0.008) but did not differ from each other. The final body weight (FBW) was greater in the DH and SH groups than in the HH group (*p* < 0.001). DMI was also greater in the DH and SH groups, resulting in a lower F/G value than that in the HH group (*p* = 0.013).

**Table 1:** Hybridization promoted growth performance in Hu sheep.

Measurements	HH	DH	SH	SEM	*p* value
Initial body weight (IBM, kg)	23.36	23.64	24.21	0.43	0.156
Final body weight (FBW, kg)	44.33^b^	49.31^a^	49.16^a^	0.81	<0.001
Average daily gain (ADG, g/d)	223.57^b^	259.29^a^	258.66^a^	11.36	0.008
Dry matter intake (DMI, kg/d)	0.86^b^	0.88^a^	0.89^a^	0.01	0.001
Feed/gain (F/G)	7.84^a^	6.86^b^	6.90^b^	0.33	0.013

Different lower-case letters indicate significant differences between groups. HH = ♂Hu × ♀Hu; DH = ♂Poll Dorset × ♀Hu; SH = ♂Southdown × ♀Hu; SEM = standard error of the mean.

### 3.2 Hybridization increased rumen epithelial development in Hu sheep

**Figure 1:**
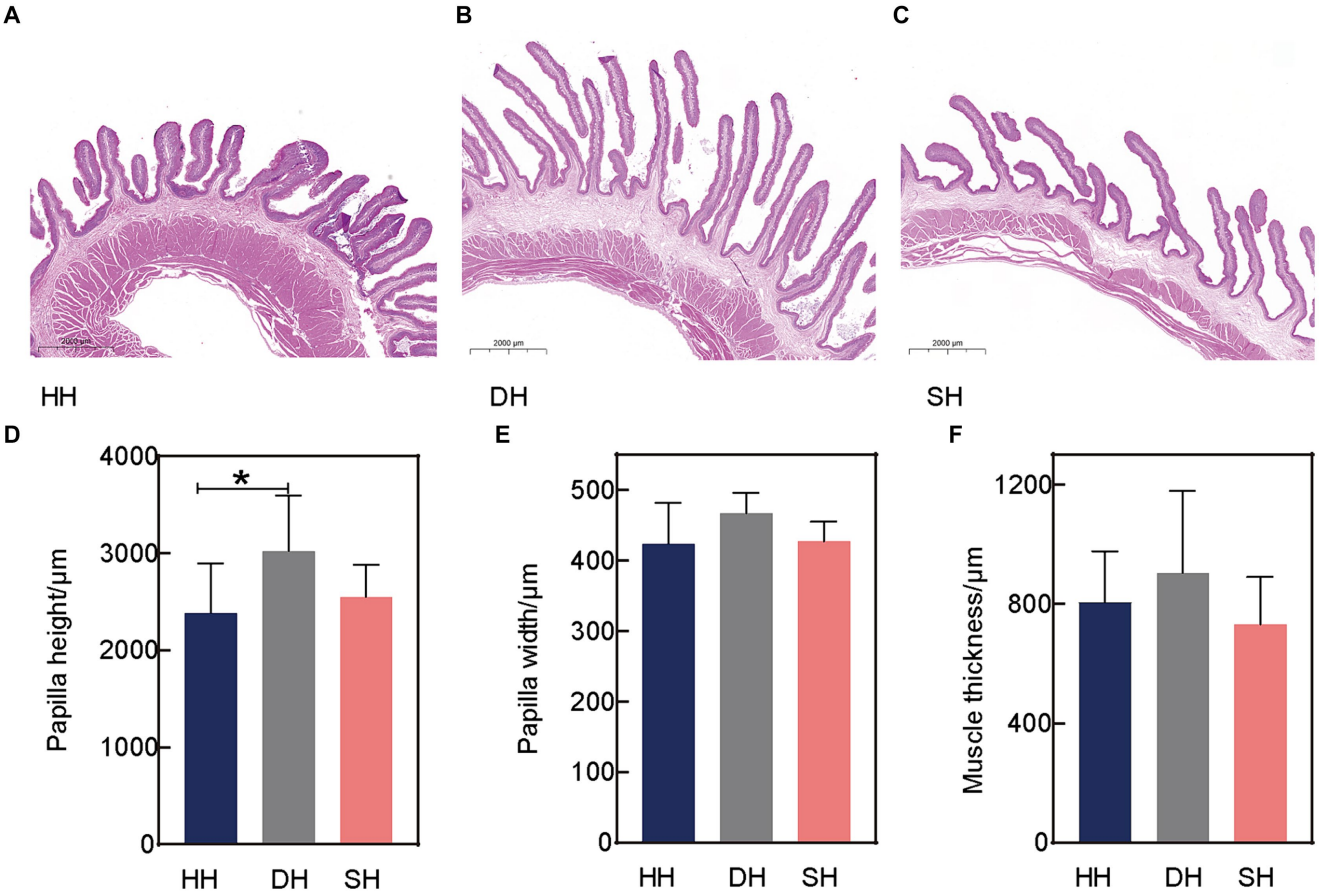
Hybridization promoted rumen epithelial development in Hu sheep. **(A)** Rumen epithelium hematoxylin and eosin (H&E) staining in the HH group. **(B)** Rumen epithelium H&E staining in the DH group. **(C)** Rumen epithelium H&E staining in the SH group. **(D)** Rumen papilla height. **(E)** Rumen papilla width. **(F)** Muscle thickness. *n* = 7 individuals/group; * indicates *p* < 0.05.

Three rumen epithelial development indices, including papilla height, papilla width, and muscle thickness, were measured using H&E staining (Figures 1A–C). Hybridization had no effect on rumen papilla width or muscle thickness (*p* > 0.128; Figures 1E,F). The largest difference was observed in papilla height, the DH group exhibited a greater height than the HH group (*p* = 0.046; Figure 1D).

### 3.3 Hybridization altered rumen fermentation parameters in Hu sheep

**Figure 2:**
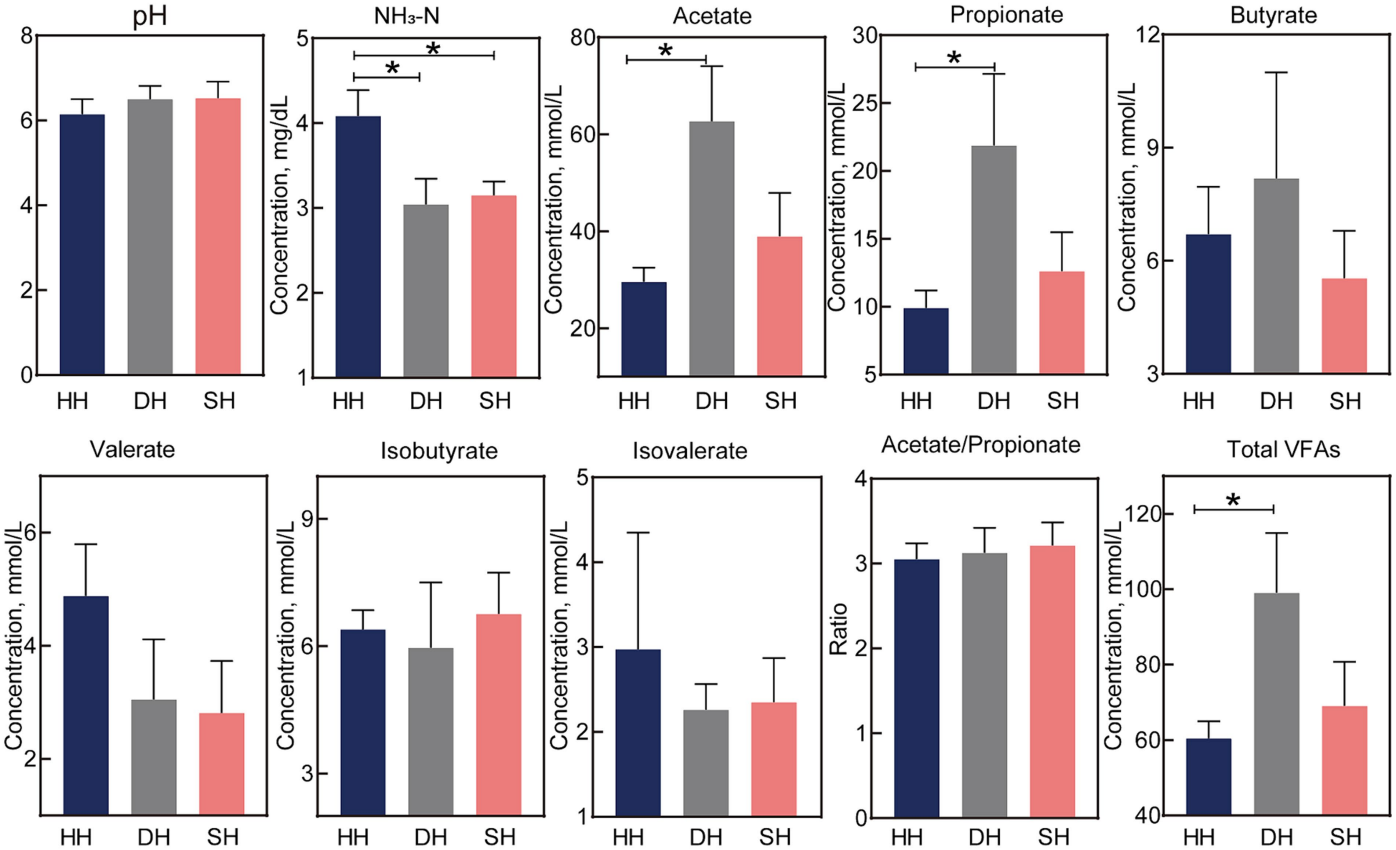
Hybridization altered rumen fermentation parameters in Hu sheep. *n* = 7 individuals/group; * indicates *p* < 0.05.

The NH_3_-N concentration was significantly lower in the SH and DH groups than in the HH group (*p* = 0.023; Figure 2). Acetate and propionate concentrations in the DH group were significantly higher than those in the HH group (*p* < 0.041), resulting in higher total VFAs (*p* = 0.048). The pH value as well as butyrate, valerate, isobutyrate, isovalerate, and acetate/propionate concentrations remained unchanged.

### 3.4 Profiling of the rumen metagenome

**Figure 3:**
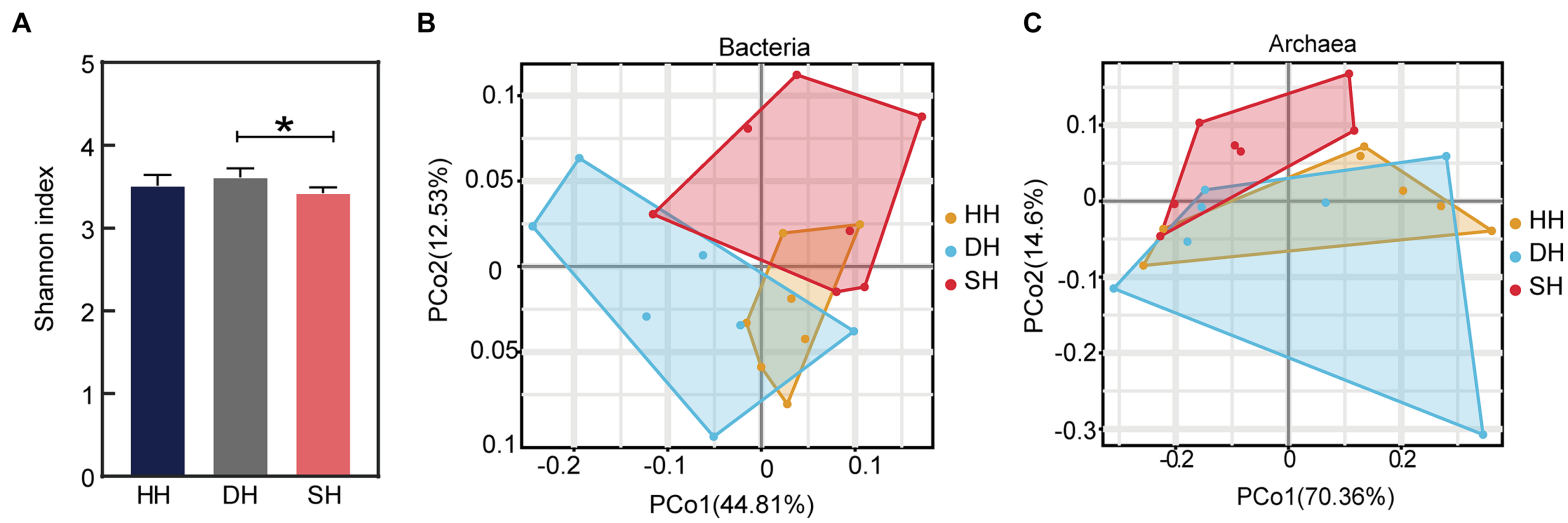
Alpha diversity index and microbial compositional profiles of rumen sample principal coordinate analysis (PCoA). **(A)** Alpha diversity as presented by the Shannon index. **(B)** PCoA based on bacterial domain. **(C)** PCoA based on archaeal domain. *n* = 7 individuals/group; * indicates *p* < 0.05.

Metagenome sequencing generated a total of 1,443,800,424 raw reads, with an average of 68,752,401 ± 5,953,935 reads (mean ± SD) per sample. After quality control and removing host genes, a total of 1,284,320,034 reads were retained, or 61,158,097 ± 5,264,516 per sample. After assembly, a total of 19,962,258 contigs were generated (with an average N50 length of 825 ± 60), with 950,584 ± 91,196 per sample (Supplementary Table S2). The results indicated that the sequencing data were credible and could be used for subsequent bioinformatics investigations. The α-diversity analysis using Kruskal–Wallis Test revealed that the Shannon index was significantly increased in the DH group compared to that in the SH group (*p* = 0.016; Figure 3A), while the Ace and Simpson indices did not differ among the three groups (Supplementary Table S3). The rumen metagenome comprised 97.18% bacteria (591,631,456 sequences), 1.60% archaea (9,758,146 sequences), 0.43% eukaryotes (2,601,458 sequences), and 0.78% viruses (4,731,066 sequences). Principal coordinate analysis (PCoA) at the domain level revealed a separation between the HH and DH groups and HH and SH groups based on bacteria (Figure 3B), as well as between the SH and HH groups and SH and DH groups based on archaea (Figure 3C), no separation was observed based on eukaryotes or viruses (Supplementary Figure S1). Therefore, the comparative analysis of rumen microbial taxa among the three groups focused only on bacteria and archaea.

### 3.5 Compositional profiles of the rumen microbiome and taxonomic differences

The dominant bacterial phyla in the rumen were Bacteroidetes (HH: 44.39%; DH: 44.87%; SH: 45.53%) and Firmicutes (HH: 42.72%; DH: 40.33%; SH: 43.24%), followed by Spirochaetes (HH: 2.81%; DH: 4.64%; SH: 2.55%); however, no significant differences were found among the three groups (*p* > 0.05; Supplementary Figure S2A). A total of 5,413 genera were identified. Among them, *unclassified o Bacteroidales* (HH: 19.53%; DH: 14.50%; SH: 20.21%), *Prevotella* (HH: 14.31%; DH: 19.93%; SH: 14.87%), and *unclassified c Clostridia* (HH: 8.33%; DH: 7.03%; SH: 8.83%) were the predominant bacteria in the rumen samples (Supplementary Figure S2B). A total of 20,943 species were found, among which the dominant bacteria were the *Bacteroidales bacterium* (HH: 19.35%; DH: 14.42%; SH: 20.00%), *Prevotella* sp. (HH: 12.37%; DH: 16.29%; SH: 12.71%) and *Clostridia bacterium* (HH: 8.33%; DH: 7.03%; SH: 8.83%) (Supplementary Figure S2C).

**Figure 4:**
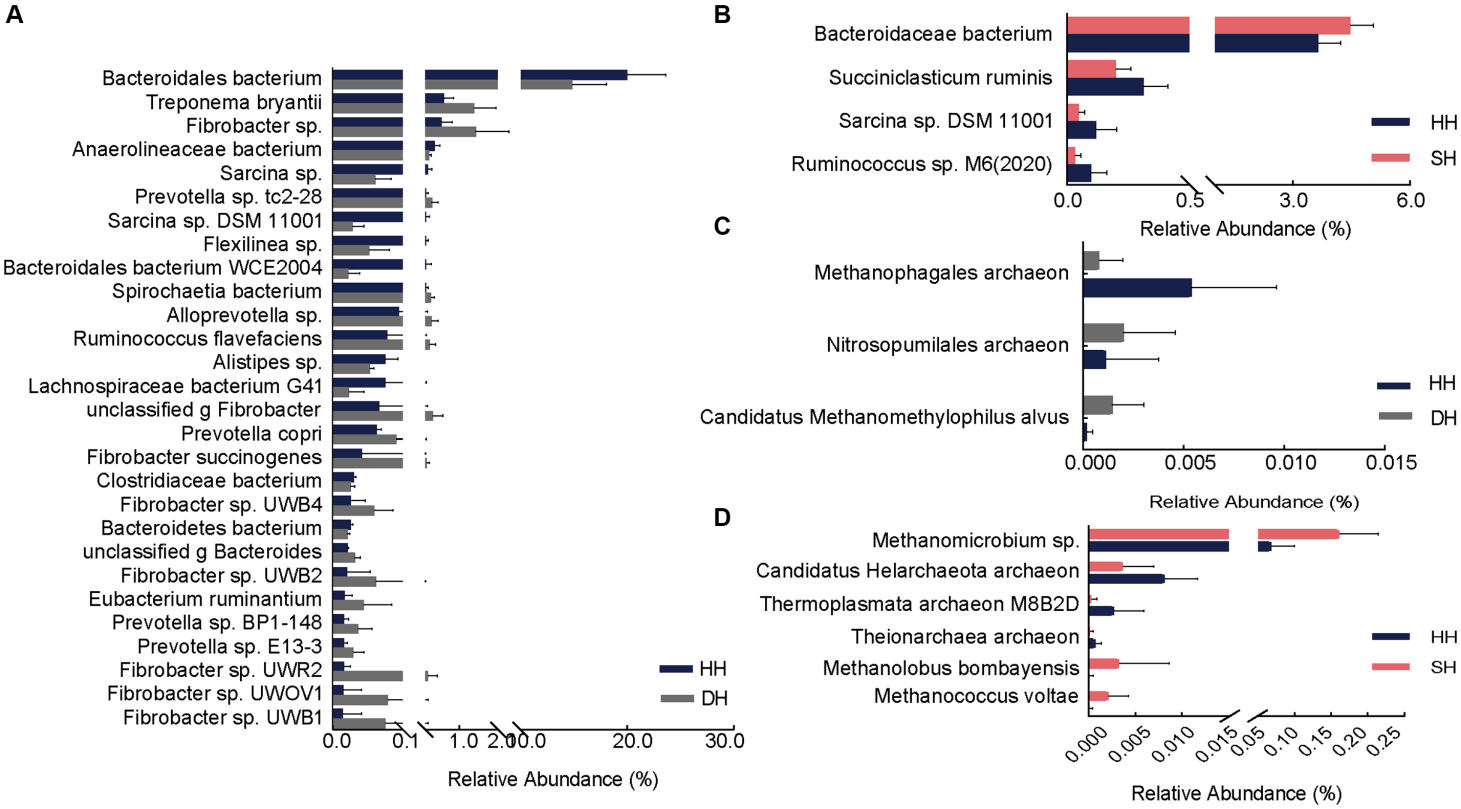
Differential rumen bacterial and archaeal species. **(A)** Significantly different bacterial species between the HH and DH groups. **(B)** Significantly different bacterial species between the HH and SH groups. **(C)** Significantly different archaeal species between the HH and DH groups. **(D)** Significantly different archaeal species between the HH and SH groups. *n* = 7 individuals/group.

Differential analysis revealed a total of 28 significantly different bacterial species between the DH and HH groups. Among them, 18 exhibited significantly higher abundances in the DH group. The relative abundances of *Treponema bryantii*, *Fibrobacter* sp., *Prevotella* sp. *tc2-28*, *Spirochaetia bacterium*, and *Alloprevotella* sp. were the top five most significantly increased. Additionally, the relative abundances of *Prevotella copri*, *Prevotella* sp. *BP1-148*, *Prevotella* sp. *E13-3*, *Fibrobacter succinogenes*, *Fibrobacter* sp. *UWB4*, *Fibrobacter* sp. *UWB2*, *Fibrobacter* sp. *UWR2*, *Fibrobacter* sp. *UWOV1*, and *Fibrobacter* sp. *UWB1* was also significantly increased in the DH group. Ten bacteria were significantly enriched in the HH group, mainly *Bacteroidales bacterium, Anaerolineaceae bacterium, Sarcina* sp., *Sarcina* sp. *DSM 11001*, and *Flexilinea* sp. (Figure 4A). Compared to levels in the HH group, only one bacterium (*Bacteroidaceae bacterium*) was significantly enriched in the SH group, whereas three (*Succiniclasticum ruminis*, *Sarcina* sp. *DSM 11001*, and *Ruminococcus* sp. M6(2020)) were significantly more abundant in the HH group (Figure 4B). When compared to levels in the DH group, 19 bacterial abundances were significantly different, including six (*Bacteroidales bacterium*, *Pyramidobacter* sp., *Clostridiaceae bacterium*, *Bacteroidales bacterium WCE2004*, *Bacteroidetes bacterium*, and *unclassified p Firmicutes*) with higher abundances in the SH group and 13 (*Treponema* sp., *Acidaminococcaceae bacterium*, *Fibrobacter* sp., *Treponema bryantii*, *Schwartzia* sp., *Prevotella* sp.*tc2-28*, *Spirochaetia bacterium*, *Schwartzia succinivorans*, *Fibrobacter succinogenes*, *Fibrobacter* sp. *UWB2*, *Fibrobacter* sp. *UWB3*, *Eubacterium ruminantium*, and *Fibrobacter* sp. *UWB12*) with higher abundances in the DH group (Supplementary Figure S3A).

At the species level, the abundance of two archaeal species (*Nitrosopumilales archaeon* and *Candidatus Methanomethylophilus alvus*) was significantly enriched and one (*Methanophagales archaeon*) significantly decreased in the DH group compared to levels in the HH group (Figure 4C). The abundance of three archaea (*Methanomicrobium* sp., *Methanolobus bombayensis*, and *Methanococcus voltae*) was significantly enriched and three (*Candidatus Helarchaeota archaeon*, *Thermoplasmata archaeon M8B2D*, and *Theionarchaea archaeon*) decreased in the SH group compared to levels in the HH group (Figure 4D). When compared with levels in the DH group, the abundances of *Methanocorpusculum* sp. and *Candidatus Altiarchaeales archaeon WOR SM1* 86-2 were significantly higher, whereas the abundances of *Candidatus Methanomethylophilaceae archaeon*, *Candidatus Methanomethylophilus* sp., *Methanomicrobium* sp., *Methanobacterium* sp. *ERen5*, *unclassified g Methanobacterium*, and *Methanolobus bombayensis* were significantly lower than those in the SH group (Supplementary Figure S3B).

### 3.6 Functional profiles and differences of the rumen microbiome

**Figure 5:**
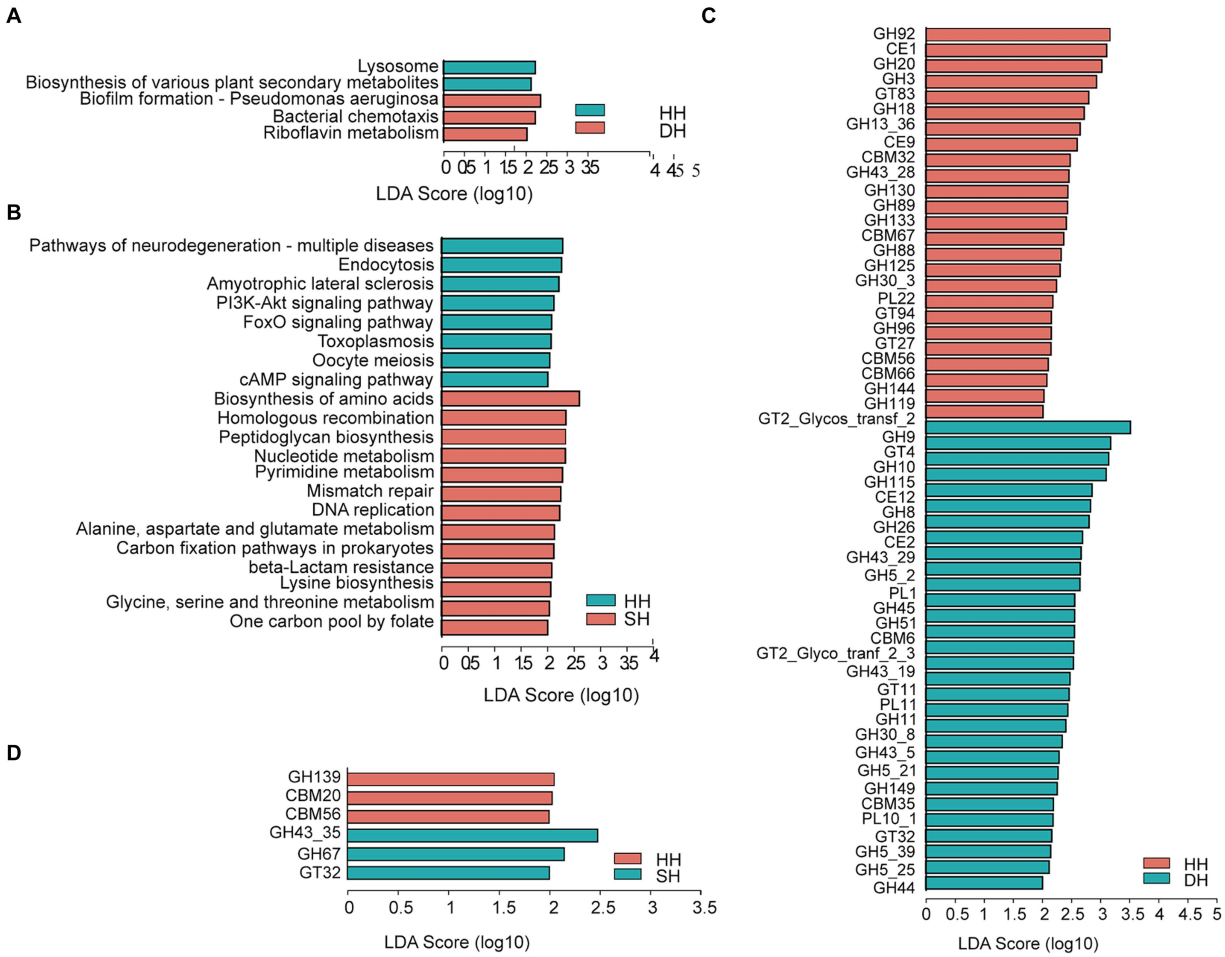
Differential rumen microbiome functions. **(A)** Significantly differential KEGG functions at the third level between the HH and DH groups. **(B)** Significantly differential KEGG functions at the third level between the HH and SH groups. **(C)** Significantly differential CAZy functions at the family level between the HH and DH groups. **(D)** Significantly differential CAZy functions at the family level between the HH and SH groups. Significant differences were tested by linear discriminant analysis effect size (LEfSe) analysis, with linear discriminant analysis (LDA) score > 2 and *p* < 0.05. *n* = 7 individuals/group.

The functions of the rumen microbiome were determined using KEGG profiles and genes encoding CAZymes. For KEGG profiles, a total of six pathways were annotated at the first level among the three groups, including “metabolism” (HH: 49.78%; DH: 50.16%; SH: 50.85%), “genetic information processing” (HH: 16.78%; DH: 16.71%; SH: 17.05%), “environmental information processing” (HH: 12.92%; DH: 12.98%; SH: 12.77%), “cellular processes” (HH: 9.71%; DH: 9.68%; SH: 9.42%), “human diseases” (HH: 6.67%; DH: 6.47%; SH: 6.22%) and “organismal systems” (HH: 4.15%; DH: 4.00%; SH: 3.70%). At the second level, we obtained only one profile, “metabolism of cofactors and vitamins,” that was significantly enriched in the DH group compared to level in the HH groups (Supplementary Figure S4A). Nine KEGG profiles were significantly enriched in the SH group compared to levels in the HH group, among them, “replication and repair,” “nucleotide metabolism,” and “biosynthesis of other secondary metabolites” were the most dominant (Supplementary Figure S4B). Seven KEGG profiles were significantly different between the DH and SH groups. “glycan biosynthesis and metabolism” and “nucleotide metabolism” were the most enriched in the SH group, while “signal transduction” was enriched in the DH group (Supplementary Figure S4C). When comparing the identified KEGG pathways at the third level, “biofilm formation—pseudomonas aeruginosa,” “bacterial chemotaxis,” and “riboflavin metabolism” were significantly enriched in the DH group, while the “lysosome” and “biosynthesis of various plant secondary metabolites” pathways were significantly decreased compared to levels in the HH group (Figure 5A). A total of 21 differential pathways were identified in the HH and SH groups. Among them, 13 were significantly enriched in the SH group, mainly including “biosynthesis of amino acids,” “homologous recombination,” “peptidoglycan biosynthesis,” “nucleotide metabolism,” “pyrimidine metabolism,” “mismatch repair,” “DNA replication,” “alanine, aspartate and glutamate metabolism,” “carbon fixation pathways in prokaryotes,” “beta-lactam resistance,” “lysine biosynthesis,” “glycine, serine and threonine metabolism,” and “one carbon pool by folate”; whereas eight were significantly enriched in the HH group (Figure 5B). Twenty differential pathways were identified in the DH and SH groups. Among them, five were significantly enriched in the DH group and 15 in the SH group. “Carbon metabolism,” “aminoacyl-tRNA biosynthesis,” “nucleotide metabolism,” “pyrimidine metabolism,” and “other glycan degradation” were the top five pathways the most significantly enriched in the SH group (Supplementary Figure S5A).

For the CAZyme profiles, 55 differentially expressed genes encoding CAZymes were identified between the HH and DH groups at the family level (Figure 5C). Among them, 23 involved in the breakdown of carbohydrates (including cellulose, hemicellulose, starch, protein, and lignin) were enriched in the DH group (2 carbohydrate esterases [CEs], 18 glycoside hydrolases [GHs], and 3 polysaccharide lyases [PLs]), and 18 in the HH group (2 CEs, 15 GHs, and 1 PL). Five glycosyltransferases (GTs) involved in carbohydrate synthesis were enriched in the DH group, while three were enriched in the HH group. Several non-catalytic CAZymes were involved in the degradation of complex carbohydrates as parts of carbohydrate-binding modules (CBMs), including two enriched in the DH group and four in the HH group. A total of six differentially expressed genes encoding CAZymes were observed between the HH and SH groups (Figure 5D). Among these, 2 GHs and 1 GT were enriched in the SH group, whereas 1 GH and 2 CBMs were enriched in the HH group. We obtained 41 differential genes encoding CAZymes between the DH and SH groups (Supplementary Figure S5B). Among those involved in carbohydrate breakdown, 21 were enriched in the SH group (2 CEs, 15 GHs, 2 GLs, and 2 PLs), whereas 12 were enriched in the DH group (2 CEs, 8 GHs, and 2 PLs). Additionally, 3 GTs were enriched in the DH group.

### 3.7 Correlation analysis between phenotype and microbiome

**Figure 6:**
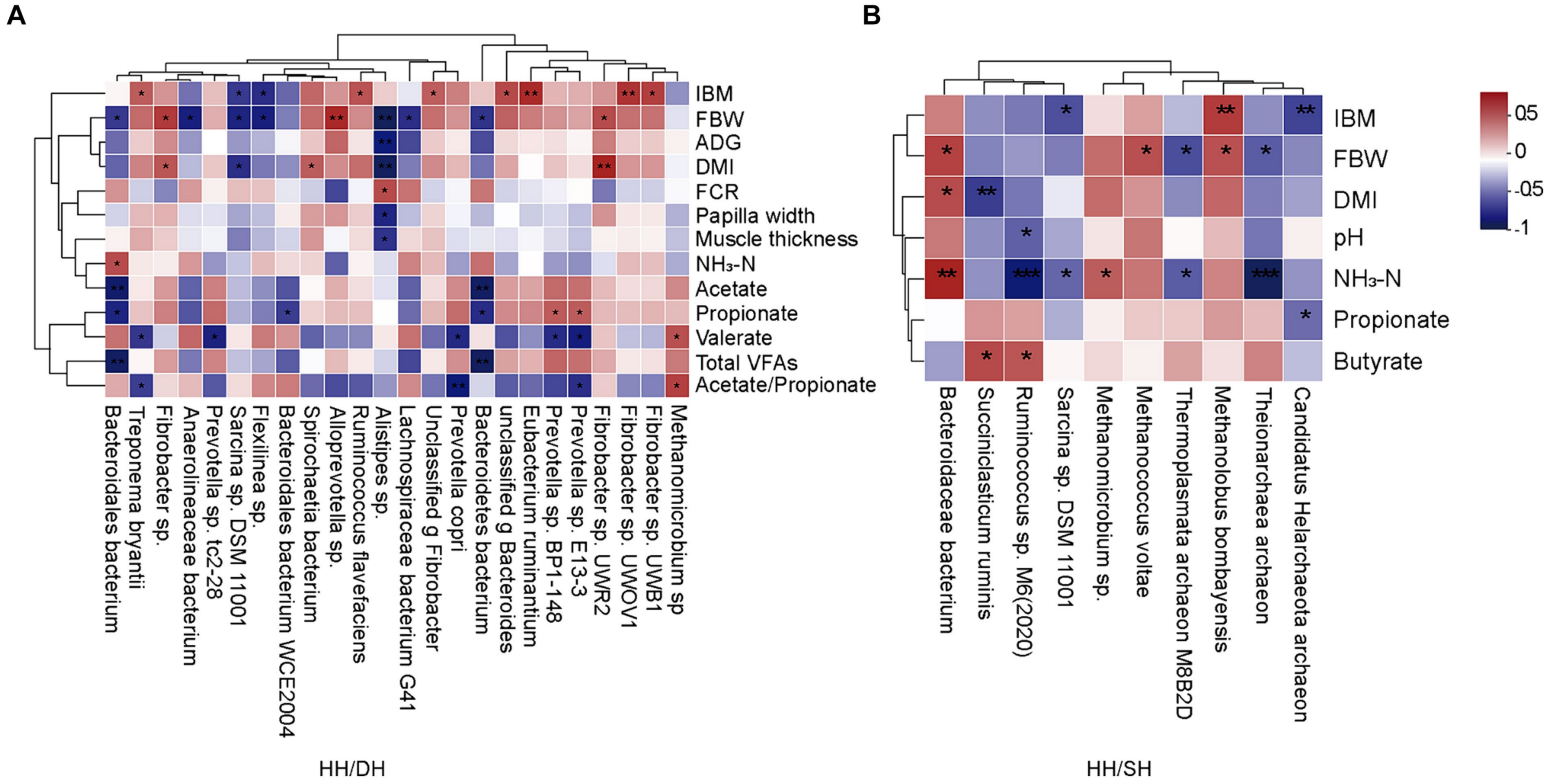
Correlation analysis between growth performance, rumen epithelial parameters, fermentation parameters, and significantly different microbiomes. (|*r*| > 0.5). **(A)** Between the HH and DH groups. **(B)** Between the HH and SH groups. *n* = 7 individuals/group. *p < 0.05, **p < 0.01, and ***p < 0.001.

Spearman’s correlation coefficient analysis identified correlations between growth performance, rumen epithelial parameters, fermentation parameters, and significantly different microbiomes (|*r*| > 0.5; *p* < 0.05). When we compared the HH and DH groups, the relative abundance of *Alloprevotella* sp. was positively correlated with FBW, as well as the relative abundance of *Fibrobacter* sp. *UWR2* and *Fibrobacter* sp. were positively correlated with DMI, and the relative abundance of *Alistipes* sp. was negatively correlated with FBW, ADG, and DMI (Figure 6A). When we compared the HH and SH groups, the relative abundance of *Bacteroidaceae bacterium* was positively correlated with FBW and DMI; however, the relative abundances of *Ruminococcus* sp. M6(2020) and *Theionarchaea archaeon* were negatively correlated with NH_3_-N concentration (Figure 6B). When we compared the DH and SH groups, we found that *Pyramidobacter* sp. was negatively correlated to acetate, propionate, and butyrate concentrations, the relative abundances of *Candidatus Methanomethylophilus* sp. and *Candidatus Methanomethylophilaceae archaeon* were negatively related to acetate, propionate, and total VFA concentrations (Supplementary Figure S6).

### 3.8 Rumen metabolome analysis

**Figure 7:**
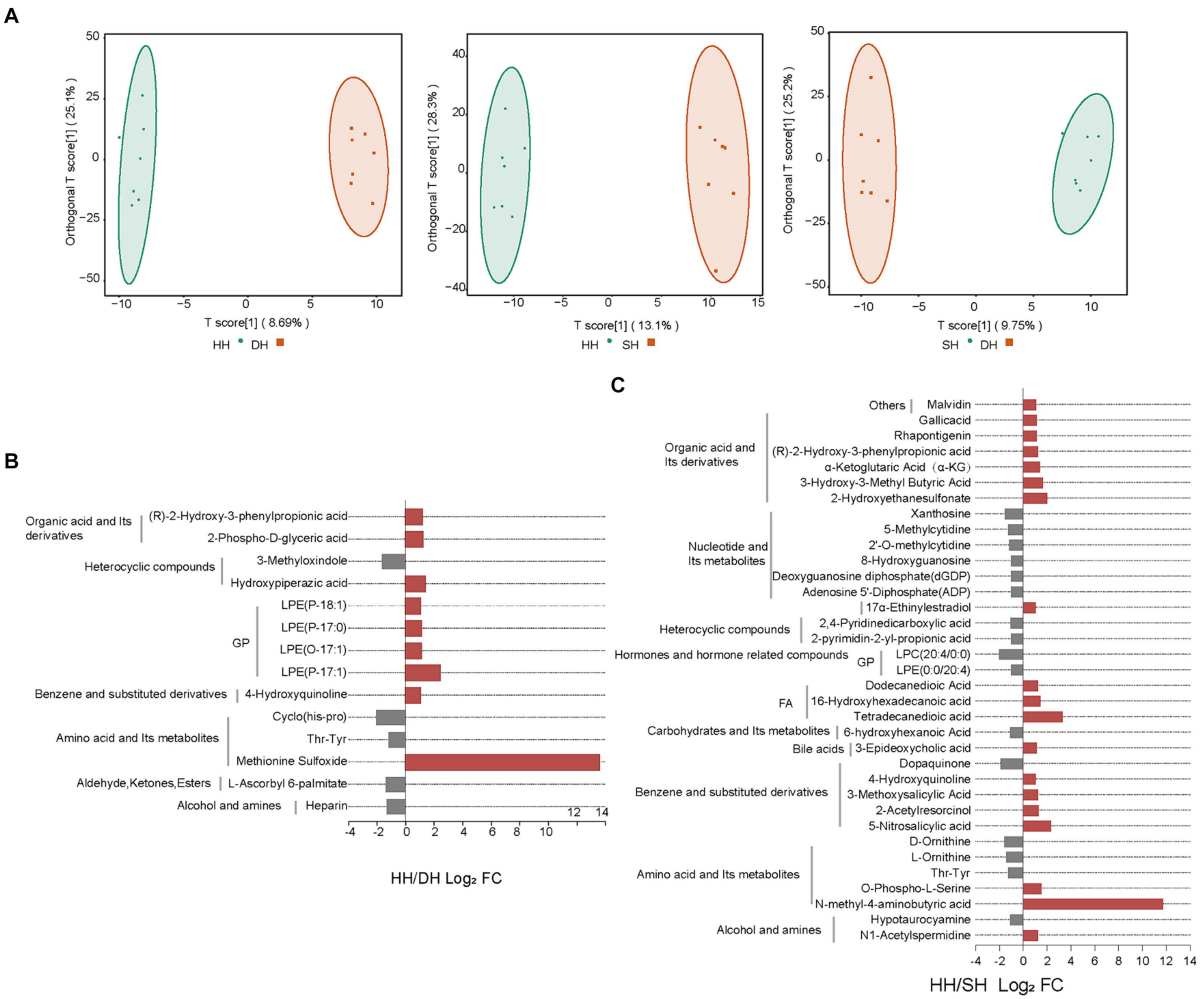
Change in rumen content metabolite levels. **(A)** OPLS-DA analysis. **(B)** Significantly differential metabolites between the HH and DH groups. **(C)** Significantly differential metabolites between the HH and SH groups. *n* = 7 individuals/group.

In total, 947 metabolites were identified in the 21 rumen samples. The orthogonal projections to latent structures-discriminant analysis (OPLS-DA) score plots showed good separation of metabolites between the HH and DH (*R*^2^*X* = 0.419, *R*^2^*Y* = 0.994, *Q*^2^ = 0.345), HH and SH (*R*^2^*X* = 0.495, *R*^2^*Y* = 0.992, *Q*^2^ = 0.580), and DH and SH (*R*^2^*X* = 0.451, *R*^2^*Y* = 0.991, *Q*^2^ = 0.315) groups (Figure 7A). After screening the relative concentrations of rumen metabolites by FC (FC ≥ 2 and FC ≤ 0.5) and VIP (VIP ≥ 1), we obtained 14 metabolites significantly differently enriched in the HH and DH groups, including nine upregulated and five downregulated metabolites (Figure 7B). Levels of lysophosphatidylethanolamine (LPE) (P-18:1), (P-17:0), (O-17:1), (P-17:1), and methionine sulfoxide were significantly increased in the DH group, while thr-tyr, cyclo (his-pro) and heparin was significantly decreased. Interestingly, methionine sulfoxide was only present in the DH group. A total of 35 metabolites were significantly differently enriched between the HH and SH groups, with19 upregulated and 16 downregulated (Figure 7C). Levels of 6 from organic acid and its derivatives, 3 from fatty acyls (FA), and 2 from amino acid and its derivatives were significantly increased in the SH group, while 6 from nucleotides and their metabolites, 2 from heterocyclic compounds, 3 from amino acids and its derivatives were significantly decreased. N-methyl-4-aminobutyric acid was identified only in the SH group. Between the DH and SH groups, 21 metabolites with significant differences were observed, including 3 upregulated and 18 downregulated. Levels of 3-hydroxy-3-methyl butyric acid, 7-ketocholesterol, and lysophosphatidylcholine (LPC) (14:0/0:0) were significantly increased, while 4 from nucleotides and their metabolites, 10 from glycerophospholipids (GP), 2 from alcohol and amines metabolites were significantly decreased (Supplementary Figure S7).

**Figure 8:**
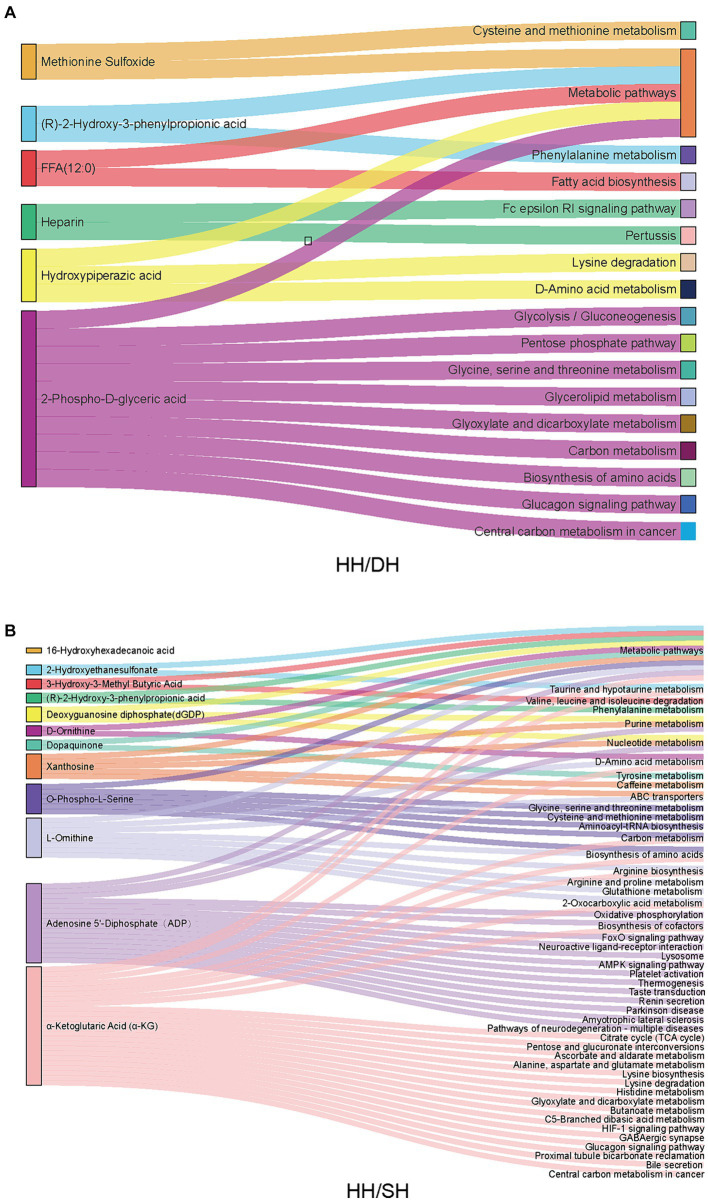
Metabolic pathway enrichment analysis. **(A)** Between the HH and DH groups. **(B)** Between the HH and SH groups. *n* = 7 individuals/group.

KEGG pathway analysis revealed that of these differential metabolites, six were annotated to 16 pathways between the HH and DH groups (Figure 8A). “Cysteine and methionine metabolism,” “phenylalanine metabolism,” “glycine, serine, and threonine metabolism,” “biosynthesis of amino acids,” “glycolysis/gluconeogenesis,” and “pentose phosphate pathway” were significantly enriched in the DH group, however, “Fc epsilon RI signaling pathway” was enriched in the HH group. Twelve metabolites were annotated in 48 pathways between the HH and SH groups (Figure 8B). Among them, relating to amino acid metabolism, bile acid metabolism and tricarboxylic acid cycle pathways were mainly enriched in the SH group, for example, “phenylalanine metabolism,” “histidine metabolism,” “glycine, serine and threonine metabolism,” “taurine and hypo taurine metabolism,” “bile secretion,” and “citrate cycle (TCA cycle).” The HH group enriched nucleotide metabolism and ABC transporters related to purine metabolism. Seven metabolites were annotated in 12 pathways between the DH and SH groups (Supplementary Figure S8). “Fat digestion and absorption,” “glycerolipid metabolism,” and “vitamin digestion and absorption” were mainly enriched in the DH group, while “valine, leucine and isoleucine degradation” and “choline metabolism in cancer” were mainly enriched in the SH group.

### 3.9 Microbiome-related metabolite analysis

**Figure 9:**
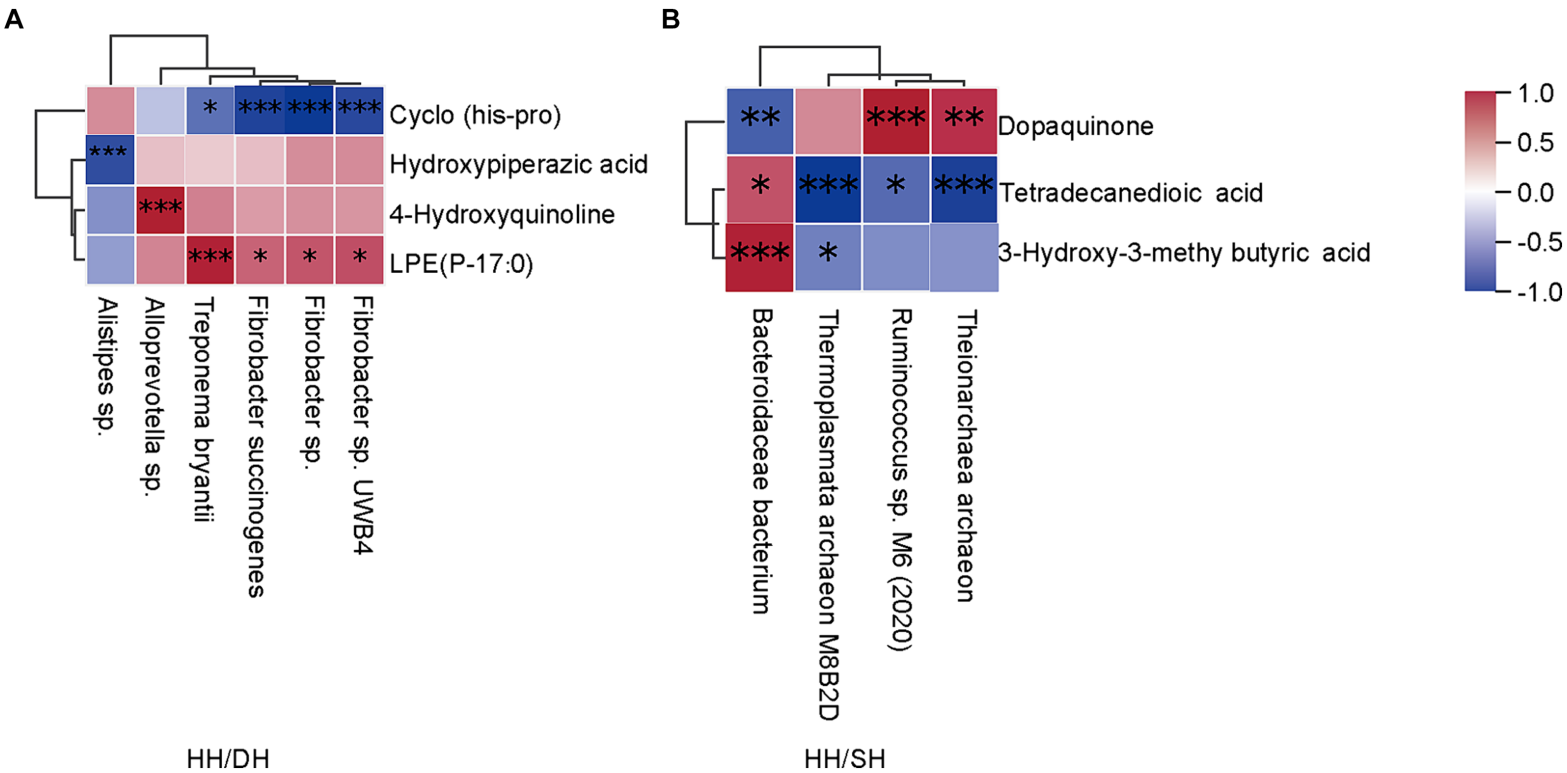
Correlation analysis between microbiome and metabolite profiles in rumen samples. (|*r*| > 0.8). **(A)** Between the HH and DH groups. **(B)** Between the HH and SH groups. *n* = 7 individuals/group. *p < 0.05, **p < 0.01, and ***p < 0.001.

Spearman’s correlation coefficient analysis identified correlations between differential microbiomes and metabolites (|*r*| > 0.8; *p* < 0.05). *Treponema bryantii*, *Fibrobacter succinogenes*, *Fibrobacter* sp. *UWB4* and *Fibrobacter* sp. levels were positively correlated with LPE (P-17:0); *Alloprevotella* sp.was positively correlated with 4-hydroxyquinoline; and *Alistipes* sp. was negatively correlated with hydroxypiperazic acid when we compared the HH and DH groups (Figure 9A). *Bacteroidales bacterium* and *Ruminococcus* sp. M6 (2020) were positively correlated with 3-hydroxy-3-methyl butyric acid and dopaquinone, respectively; whereas *Theionarchaea archaeon* and *Thermoplasmata archaeon M8B2D* were negatively correlated with tetradecanedioic acid in the HH and SH groups (Figure 9B). *Candidatus Altiarchaeales archaeon WOR SM1* 86-2 was positively correlated with LPE (16:0/0:0), LPE (0:0/16:0), LPE (O-18:2), phosphatidylcholine (PC) (O-1:0/O-16:0), lysophosphatidylserine (LPS) (20:0), lysophosphatidic acid (LPA) (22:6); 7-ketocholesterol was positively correlated with *Candidatus Methanomethylophilaceae archaeon*, *Candidatus Methanomethylophilus* sp., and *Bacteroidales bacterium* between the DH and SH groups (Supplementary Figure S8).

## 4 Discussion

From the perspective of genetics and breeding, introducing a favorable exogenous gene may be a better choice for improving the low to moderate heritability of traits (28). In this study, we screened Poll Dorset and Southdown sheep as sires to improve the growth performance of Hu sheep, focusing on how hybridization affects growth performance and investigating the regulation mechanism of the rumen microbiome and its metabolites.

Hybridization results in heterosis and improves animal growth. As expected, our data showed that hybridization increased DMI and ADG, resulting in higher FBW and lower F/G. Our previous study indicated that F_1_ hybrid offspring grew faster and became larger than did Hu sheep, particularly during the later periods (29). Another study on a three-way cross system between Angus, Qaidam, and yak indicated that the F_2_ offsprings were all significantly better than those of the yak and 1/2 yak (28). These results suggest the importance of hybridization for improving growth performance of animals.

Rumen microecology is closely associated with animal growth. In this study, hybridization significantly changed the rumen fermentation pattern in sheep, especially the NH_3_-N concentration. NH_3_-N is mainly derived from microbe-mediated amino acid deamination and non-protein nitrogen hydrolysis in the rumen (30). The rumen microbiome utilizes NH_3_-N to provide primary protein synthesis precursors for the host. NH_3_-N also has a feedback effect on rumen microbiota structure and epithelial function, affecting rumen fermentation and host health (31). The NH_3_-N concentration was significantly lower in the SH and DH groups than in the HH group, indicating that the hybrid offspring had a higher utilization efficiency of nitrogen sources to synthesize bacterial proteins. VFAs are the main energy source for ruminants, which ferment plant materials into VFAs that regulate a variety of physiological functions in the rumen (32). Acetate, propionate, and butyrate are the three major VFAs and provide approximately 70% of energy requirements. Acetate concentration is mainly affected by the microbial degradation of fibrous substances. Reports have shown that rumen acetate concentration increases when ruminants are fed a high-fiber diet (33) or when the rumen microbiota is dominated by *Prevotella* (34). Of the acetate in the rumen, 67% is used for oxidative energy, while the rest is used for body fat synthesis. Propionate contributes to energy supplementation via the gluconeogenic pathway to promote glucose synthesis (35). In this study, acetate and propionate levels in the DH group significantly increased, resulting in higher total VFAs, indicating a higher energy supply to the host and improved growth performance.

The rumen epithelium is an important indicator of rumen development and is responsible for the absorption and metabolism of nutrients and microbial byproducts. Evidence has shown that VFAs are absorbed through the rumen epithelium and that their absorption capacity is mainly related to the surface area and expression of VFA transporter vectors in the epithelium (36). The rumen epithelium is abundant in mitochondria, powering epithelial metabolism; a thicker epithelium indicates more efficient feed utilization in cattle (37). Additionally, butyrate stimulates epithelial cellular proliferation (38). Our results indicate that hybridization increased papilla height, while no change was observed in papilla width or muscle thickness, which suggests an increased nutrient contact surface area to completely crush the feed to promote digestion and feed efficiency (39). Moreover, these variations in rumen epithelial physical structures are considered to potentially influence the rumen microbiota.

Using PCoA, the present study found significant differences in the rumen microbiome between the F_1_ hybrid offspring and Hu sheep. Hybridization can produce new gene flow and different gene expression patterns in offspring. Sika and elk deer have different rumen microbiota than do their hybrid offspring; pure and hybrid mice have different microbial clustering characteristics suggesting a significant effect of host genetics on the rumen microbiome that may result from vertical transmission (40–42). Moreover, we found that Bacteroidetes and Firmicutes were the dominant bacteria at the phylum level, however, hybridization had no considerable effect on their relative abundances. Evidence has shown that Bacteroidetes have low heritability estimates, while Firmicutes have moderate heritability estimates, suggesting that they are largely affected by environmental factors, such as diet (15, 43). Concurrently, the different heritability estimates indicate that host effects are not equal for different rumen microbial phylotypes. At the species level, we observed that bacteria related to fiber degradation were more abundant in the DH group, *Treponema bryantii*, *Prevotella copri*, and *Fibrobacter succinogenes* were particularly abundant. *Treponema bryantii*, when present with cellulolytic species such as *Bacteroides succinogenes*, can promote the digestion of cellulosic materials to increase feed efficiency (44). A higher abundance of *Treponema bryantii* and *Bacteroides* sp. were found in the cecum of pigs with high feed efficiency (45). *Prevotella* are considered the most important bacteria for polysaccharide degradation and fermentation based on their genomic glycoside hydrolase profiles, gene expression, and abundance in the rumen (46). *Prevotella copri* might be the keystone bacterial species associated with host feed intake and fat metabolism, and evidence shows that its abundance increases in pigs with high average daily feed intake (47) and promotes fat accumulation in pigs fed formula diets (48). Studies in humans indicate that *Prevotella copri* largely increases the microbial potential for branched-chain amino acid biosynthesis (49), which is beneficial for milk protein yield in dairy cattle and animal growth (50, 51). Moreover, *Prevotella copri* is heritable (*h*^2^ > 0.15) in the cecum and feces of pigs (52). *Fibrobacter succinogenes* is regarded as the most highly cellulolytic bacterium in ruminal microbiomes and specializes in the production of succinate, acetate, and formate (53). An increase in the *Fibrobacter succinogenes* population in the rumen is attributed to an increase in dry matter degradability (54). In the present study, the relative abundances of *Fibrobacter* sp. *UWR2* and *Fibrobacter* sp. were positively linked to DMI, suggesting their essential roles in DMI and dry matter degradability. In the SH group, the abundance of the *Bacteroidaceae bacterium* was higher than that of the HH group. *Bacteroidaceae bacterium* is also a rumen cellulolytic bacterium that can convert succinate to propionate as the sole energy source (55). Therefore, the higher abundance of polysaccharide-degrading bacteria in the rumen of hybrid sheep enhances the degradation of dietary carbohydrates, thus promoting digestive ability and growth performance. This may be attributed to host genetics because we excluded the effects of environmental factors. However, this speculation needs to be verified by monitoring the rumen microbiota of Poll Dorset and Southdown sheep. The DH group lacked archaea related to nutrient digestion and absorption, however, the abundance of methanogenic bacteria (*Methanomicrobium* sp., *Methanolobus bombayensis*, and *Methanococcus voltae*) increased in the SH group compared to levels in the HH group, and methane production is an energy-intensive process, resulting in low production performance. However, in this study, the abundance of these methanogenic bacteria was quite low. We also found that DMI and feed protein utilization efficiency in the SH group were higher than those in the HH group, which suggests that the contributions of DMI and feed protein utilization efficiency surpassed the methane production rate, which may be the reason for the FBW gain in the SH group. Moreover, Xue et al. found that some methanogenic bacteria with low abundance were enriched in the rumens of dairy cows with high milk protein yields (50); however, further studies are needed to validate our hypotheses.

KEGG functional taxonomic analysis revealed that amino acid biosynthesis and metabolism were enriched in the SH group. Metabolic products such as lysine, aspartate, glycine, alanine, glutamate, serine, and threonine are important contributors to rumen microbial protein synthesis, constituting 90% of the microbial proteins that arrive in the small intestine and provide a protein source for the host (56). Our previous study identified that the muscle protein content was higher in the SH group than in the HH group (29), which is in line with the results of the rumen microbiota. Genes encoding CAZymes involved in deconstructing carbohydrates (GHs, CEs, PLs, and CBMs) were enriched in the DH group, indicating that these sheep were more capable of degrading complex substrates to promote microbial fermentation, which is consistent with our VFA results.

In addition to affecting the composition and function of the rumen microbiome, hybridization alters rumen metabolites and metabolic pathways. First, LPEs are cell membrane components and are the second most abundant *in vivo* after LPCs (57, 58). LPEs reportedly induce the activation of mitogen-activated protein kinase signaling and exhibit anti-apoptotic effects in pheochromocytoma cells (59). LPE (P-18:1) has oleic acid as its acyl chain, stimulating neurite outgrowth and neuroprotective effects against glutamate-induced excitotoxicity (60). Additionally, LPEs are involved in lipid droplet formation by suppressing lipolysis and fatty acid biosynthesis, thereby promoting the absorption of lipid substances (61). This evidence indicates that LPEs enriched in the DH group benefited from fat deposition, resulting in FBW gain. Interestingly, methionine sulfoxide was only present in the DH group. The exposure of proteins to reactive oxygen species and hydrogen peroxide may lead to the oxidation of free methionine and methionine residues, forming methionine sulfoxide (62). Methionine sulfoxide levels are elevated during oxidative stress, aging, and inflammation. The results of this study imply that the sheep in the DH group had poor environmental adaptability. However, methionine sulfoxide can be diastereoselectively repaired by methionine sulfoxide reductase to produce methionine (63). Methionine augments the endogenous antioxidant capacity of rice proteins by stimulating methionine sulfoxide reductase expression and enhancing glutathione synthesis via the Nrf2-ARE pathway (64). The beneficial effects of methionine supplementation on the antioxidant activity of heat-stressed birds have been previously demonstrated (65). The present study shows that although sheep in the DH group were in a state of oxidative stress, this phenomenon can be improved by regulating the methionine sulfoxide reductase system, which will be our next research direction. N-methyl-4-aminobutyric acid was only present in the SH group. The metabolic product aminobutyric acid acts as a key inhibitory neurotransmitter of the central nervous system and functions via sedation, relieving fever and decreasing heat production (66). Aminobutyric acid reduces stress caused by various environmental conditions and improves productivity (67). For example, aminobutyric acid improves growth performance, meat quality, and feed intake by promoting the activation of characteristic enzymes and improving the absorption and immune function of the intestinal mucosa under heat-stress conditions (68, 69). The characteristic metabolites in both the DH and SH groups were related to oxidative stress, indicating that the environmental adaptability of the F_1_ generation sheep needs improvement, which we expect to occur in the F_2_ generation. α-Ketoglutaric acid is an intermediate metabolite of the tricarboxylic acid cycle and is generated from glucose and glutamine, providing substrates for the synthesis of carbohydrates, amino acids, fats, and other biomolecules (70). In this study, α-ketoglutarate was significantly increased in the SH group compared to levels in the HH group, further enhancing amino acid metabolism ability. Our results were consistent with a previous report by Kong (71), who confirmed the tricarboxylic acid cycle pathways were enhanced in the muscle of Southdown × Hu F_1_ generation sheep.

Four metabolites and six microbiomes showed regulatory relationships in growth performance between the DH and HH groups. *Alloprevotella* sp. can ferment carbohydrates to produce acetate and succinate as end-metabolites and is positively correlated with 4-hydroxyquinoline, which has antibacterial and anti-inflammatory effects that maintain rumen health (72). *Treponema bryantii*, *Fibrobacter succinogenes*, *Fibrobacter* sp., and *Fibrobacter* sp.*UWB4* are associated with fiber degradation and production of propionate, which are positively correlated with LPE (P-17:0). Propionate can promote glucose synthesis via the gluconeogenic pathway in the rumen, and glucose is a precursor for the form of glycerol and fatty acids, which further help format LPEs. LPEs originate in part from the lipids in the diet, but also from gut microbial synthesis. Phospholipid levels in the host cells of germ-free mice differ from those in the host cells of conventional mice, suggesting a relationship between gut microbes and glycerophospholipids (73). The relative abundance of *Faecalibacterium* and *Prevotella* is related to circulating lipids, particularly LEPs and phosphatidylglycerol (74); however, we found in the present study that *Fibrobacter* was also related to the levels of LPE (P-17:0). *Bacteroidales bacterium* was positively correlated with 3-hydroxy-3-methyl butyric acid between the SH and HH groups. 3-Hydroxy-3-methyl butyric acid is a leucine metabolite that stimulates the growth hormone/insulin-like growth factor-1 axis (75). These results indicate that the rumen microbiota of sheep in the DH group were mainly involved in body fat deposition by increasing DMI and dry matter degradability, whereas that in the SH group mainly participated in amino acid metabolism to provide energy. However, the causal relationship between the microbiota and metabolites requires further research. In addition, the rumen microbiomes and metabolites data of Poll Dorset and Southdown sheep should be considered and further elucidated in future studies.

## 5 Conclusion

The results of this study support the feasibility of using crossbreeding programs to promote growth and increase feed efficiency by altering the rumen microbiota and metabolites in sheep. In the DH group, *Treponema bryantii*, *Prevotella copri*, and *Fibrobacter succinogenes* levels increased, and their functions were mainly enriched at the CAZyme level, contributing to higher acetate, propionate, total VFA, and LPE levels to further promote growth. *Bacteroidaceae bacterium* was enriched in the SH group and is involved in amino acid metabolism, fulfilling the demand for ruminal microbial proteins that are utilized by hosts to promote growth. Additionally, methionine sulfoxide and N-methyl-4-aminobutyric acid were characteristic metabolites in the DH and SH groups, respectively, indicating that the F_1_ generation crossbred sheep had poor environmental adaptability. Taken together, these findings provide new ideas and nutritional strategies for the cultivation of new sheep breeds.

## Data Availability

The data presented in the study are deposited in the SRA repository, accession number PRJNA1111660.

## Footnotes

^1^http://hmmer.janelia.org/search/hmmscan

^2^http://www.cazy.org/
