# Supplementary material for: Hybridization promotes growth performance by altering rumen microbiota and metabolites in sheep
Source: Front Vet Sci. 2024 Sep 25;11:1455029. doi: 10.3389/fvets.2024.1455029 (PMC11461465; doi:10.3389/fvets.2024.1455029)
Supplement: Supplementary file 1 [file Data_Sheet_1.docx]

Supplementary Material

**Hybridization promotes growth performance by altering rumen microbiota and metabolites in sheep**

Rui Zhang^1,2,3^, Liwa Zhang^1,2,3^, Xuejiao An^1,2,3^, Jianye Li^1,2,3^, Chune Niu^1,2,3^, Jinxia Zhang^4^, Zhiguang Geng^4^, Tao Xu^1,2,3,5^, Bohui Yang^1,2,3^, Zhenfei Xu^1,2,3*^ and Yaojing Yue^1,2,3*^

*** Correspondence:** Zhenfei Xu: [236xuzhenfei@163.com](mailto:236xuzhenfei@163.com); Yaojing Yue: yueyaojing@caas.cn

**Supplementary Table 1 Ingredient composition and nutrient levels of experimental diets (Dry matter basis, %)**

| Ingredients | Contents |  | Nutrient levels | Contents |
| --- | --- | --- | --- | --- |
| Oat hay | 6.08 |  | Dry matter | 61.68 |
| *Leymus* chinensis | 13.04 |  | Crude protein | 13.19 |
| Corn silage | 13.53 |  | Acid detergent fiber | 12.75 |
| Corn | 32.78 |  | Neutral detergent fiber | 23.60 |
| Megalac^1）^ | 6.08 |  | Fat | 3.19 |
| Lamb9303^2）^ | 28.49 |  | Starch | 23.01 |
| Total | 100.00 |  | Calcium | 0.52 |
|  |  |  | Phosphorus | 0.29 |

^1）^Megalac: Rumen fatty acid calcium, Yihai Kerry, Tianjin.

^2）^Lamb9303:mainly composed of soybean oil, soybean meal, cottonseed meal, rapeseed meal, DDGS, calcium carbonate, calcium nitrogen phosphate, sodium chloride, a variety of trace elements and vitamins; its crude protein content≥32.0%, crude ash≤21.0%, calcium is1.4%~5.0%, total phosphorus≥0.60%, crude fiber≤20.0%, lysine≥1.0%, sodium chloride is 1.5%~5.0%.

**Supplementary Table 2 Summary of sequence data generated from rumen samples of 21 sheep.**

| Samples | Raw reads | Clean reads | Optimized reads | Contigs | N50(bp) | ORFs |
| --- | --- | --- | --- | --- | --- | --- |
| HH_Ru_1 | 74257598 | 73104252 | 65804476 | 1117123 | 750 | 1475820 |
| HH_Ru_2 | 67713906 | 66549308 | 61343196 | 1007432 | 743 | 1331814 |
| HH_Ru_3 | 73618966 | 72172810 | 65032060 | 1078185 | 736 | 1405145 |
| HH_Ru_4 | 66531706 | 65548664 | 60262216 | 999481 | 775 | 1323394 |
| HH_Ru_5 | 68212980 | 67092008 | 61599270 | 1026894 | 823 | 1402184 |
| HH_Ru_6 | 59569256 | 58736814 | 55121566 | 836592 | 868 | 1154957 |
| HH_Ru_7 | 68364960 | 67195502 | 60980506 | 958006 | 859 | 1307123 |
| DH_Ru_1 | 69717588 | 68395212 | 62085440 | 873968 | 906 | 1222162 |
| DH_Ru_2 | 71888104 | 70786060 | 61637696 | 856979 | 871 | 1144298 |
| DH_Ru_3 | 63946238 | 63111530 | 54084292 | 922579 | 691 | 1189153 |
| DH_Ru_4 | 67606196 | 66602462 | 60889436 | 970557 | 841 | 1320709 |
| DH_Ru_5 | 60250688 | 59338350 | 53805736 | 791398 | 849 | 1082139 |
| DH_Ru_6 | 71494148 | 70402398 | 63131720 | 858555 | 873 | 1184912 |
| DH_Ru_7 | 60650430 | 59396810 | 53447764 | 874493 | 833 | 1208529 |
| SH_Ru_1 | 67152688 | 66246672 | 59316812 | 897110 | 901 | 1250912 |
| SH_Ru_2 | 74887466 | 73746912 | 66769264 | 955681 | 901 | 1345761 |
| SH_Ru_3 | 69336868 | 68270836 | 62843150 | 1014426 | 774 | 1366001 |
| SH_Ru_4 | 63006160 | 61817010 | 56310132 | 940959 | 837 | 1293815 |
| SH_Ru_5 | 78221526 | 76670748 | 67784554 | 958618 | 860 | 1325454 |
| SH_Ru_6 | 64421324 | 63258706 | 57121970 | 893626 | 815 | 1220407 |
| SH_Ru_7 | 82951628 | 81744048 | 74948778 | 1129596 | 827 | 1553608 |
| SUM | 1443800424 | 1420187112 | 1284320034 | 19962258 | 17333 | 27108297 |
| Average | 68752401.14 | 67627957.71 | 61158096.86 | 950583.7 | 825.381 | 1290871 |
| SD | 5953935.18 | 5854461.813 | 5264515.7 | 91196.15 | 59.68038 | 115238.6 |

**Supplementary Table 3 Alpha diversity indexes estimators of sheep.**

| Estimators | HH | DH | SH | *P* value | | |
| --- | --- | --- | --- | --- | --- | --- |
|  |  |  |  | HH-SH | HH-DH | SH-DH |
| Ace | 11160.00±615.40 | 10450.00±902.40 | 10700.00±698.90 | 0.475 | 0.167 | 0.795 |
| Shannon | 3.51±0.13 | 3.61±0.11^a^ | 3.42±0.07^b^ | 0.287 | 0.424 | 0.016 |
| Simpson | 0.08±0.01 | 0.08±0.01 | 0.09±0.01 | 0.424 | 0.744 | 0.117 |


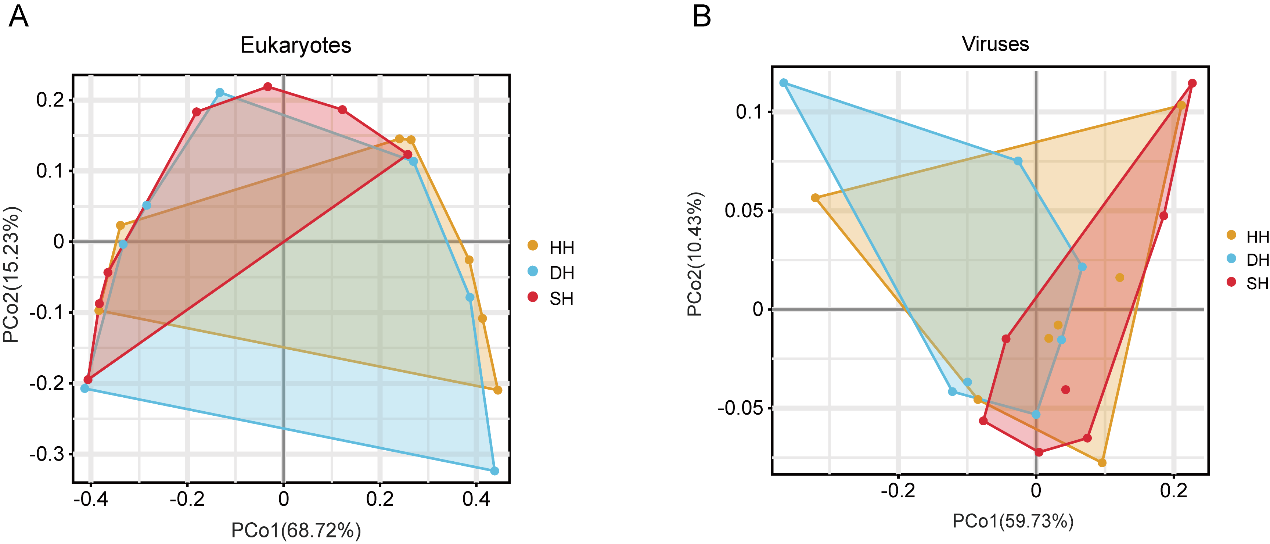


**Supplementary Figure 1** Microbial compositional profiles of rumen content samples based on domain-level using principal-coordinate analysis (PCoA). (A) Eukaryotes. (B) Viruses.


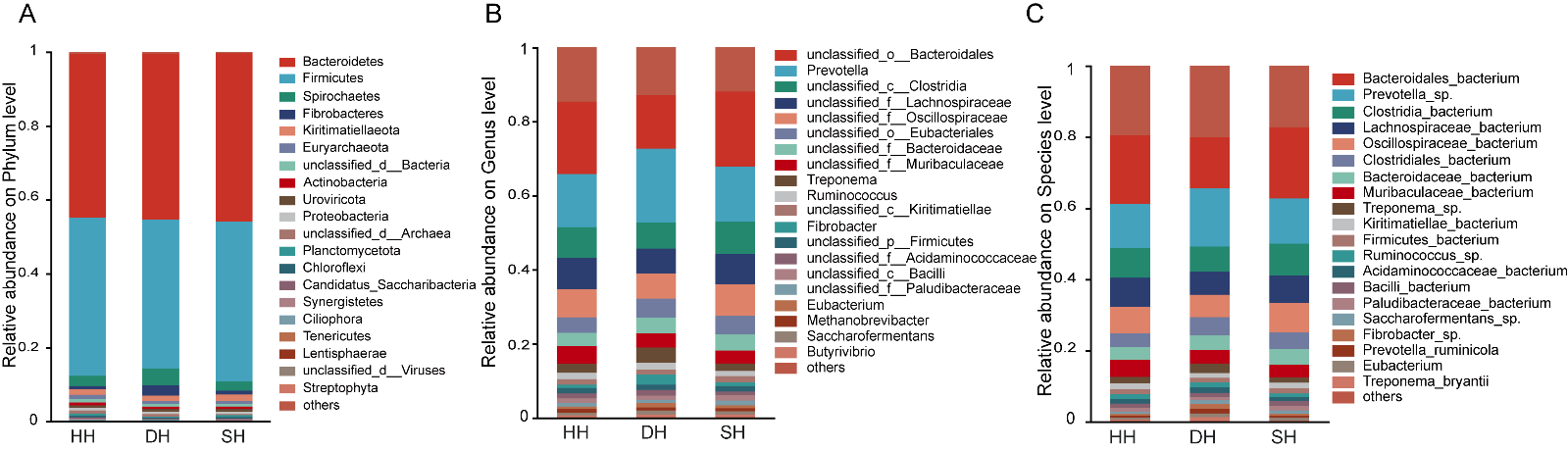


**Supplementary Figure 2** The composition of the rumen microbiota of sheep. (A) At the phylum level. (B) At the genus level. (C) At the species level.


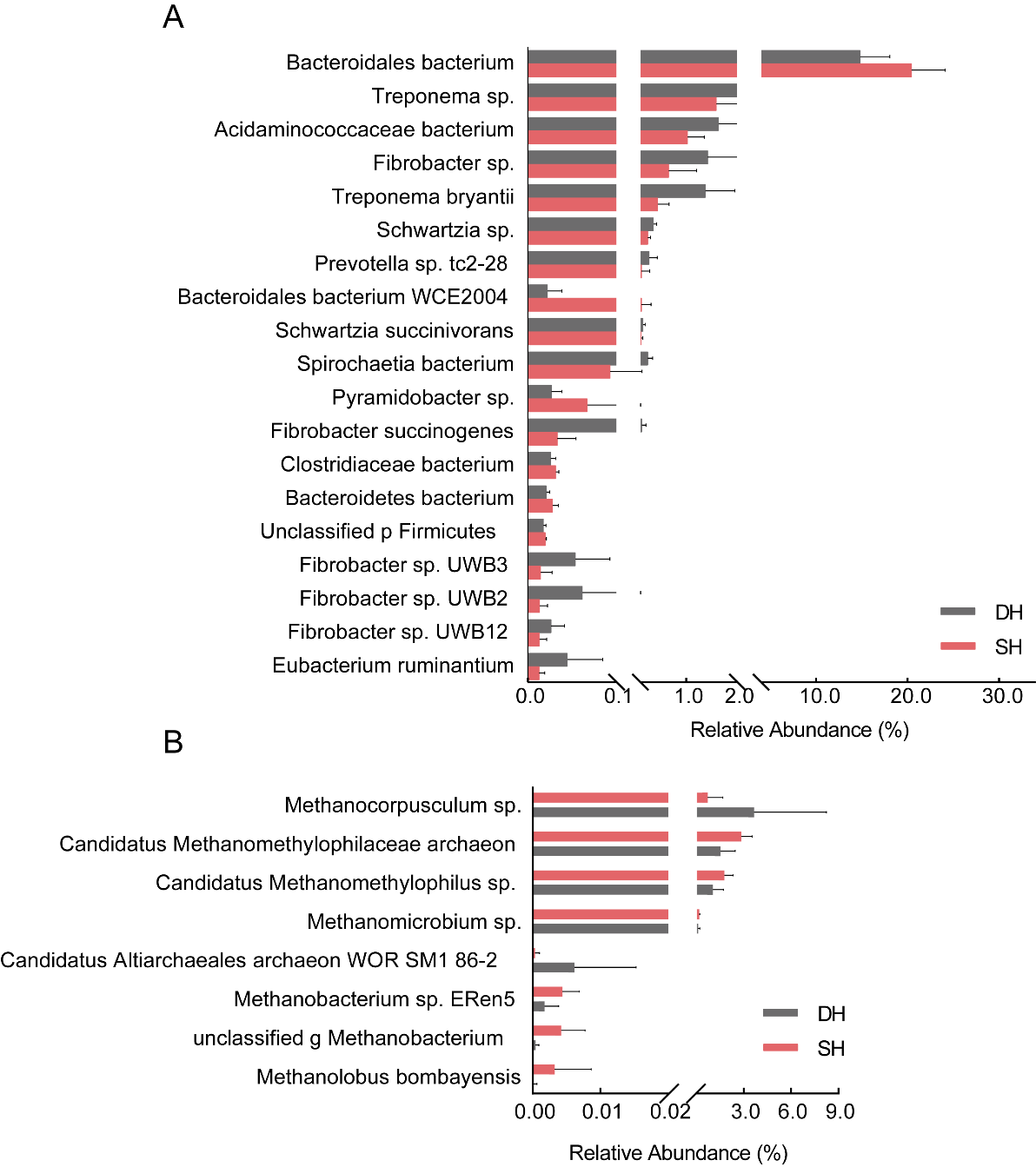


**Supplementary Figure 3** Differential rumen bacterial and archaeal species between the DH and SH groups. (A) bacterial. (B) archaeal.


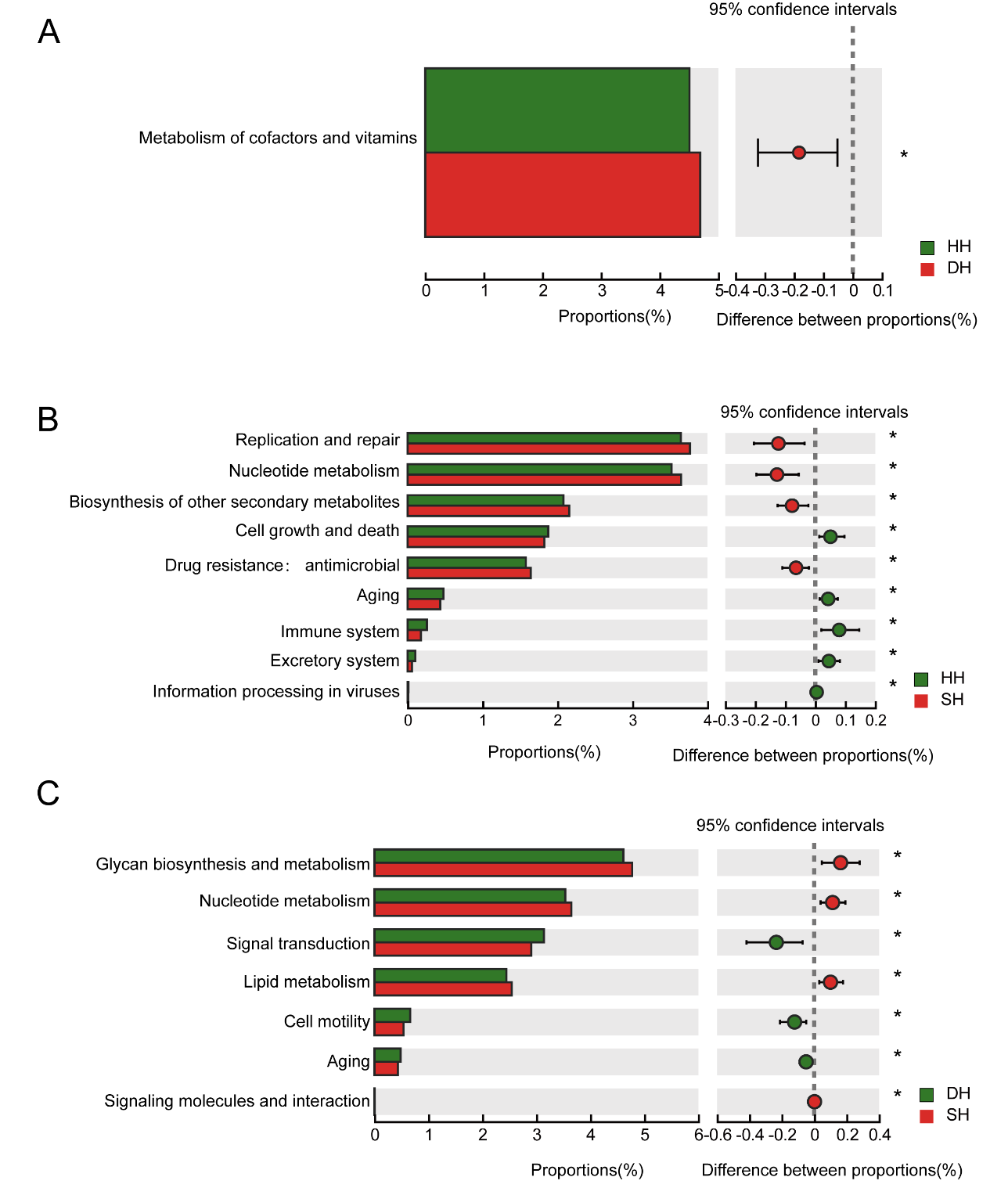


**Supplementary Figure 4** Differential rumen microbiota KEGG functions at the second-level. (A) Between the HH and DH groups. (B) Between the HH and SH groups. (C) Between the DH and SH groups.


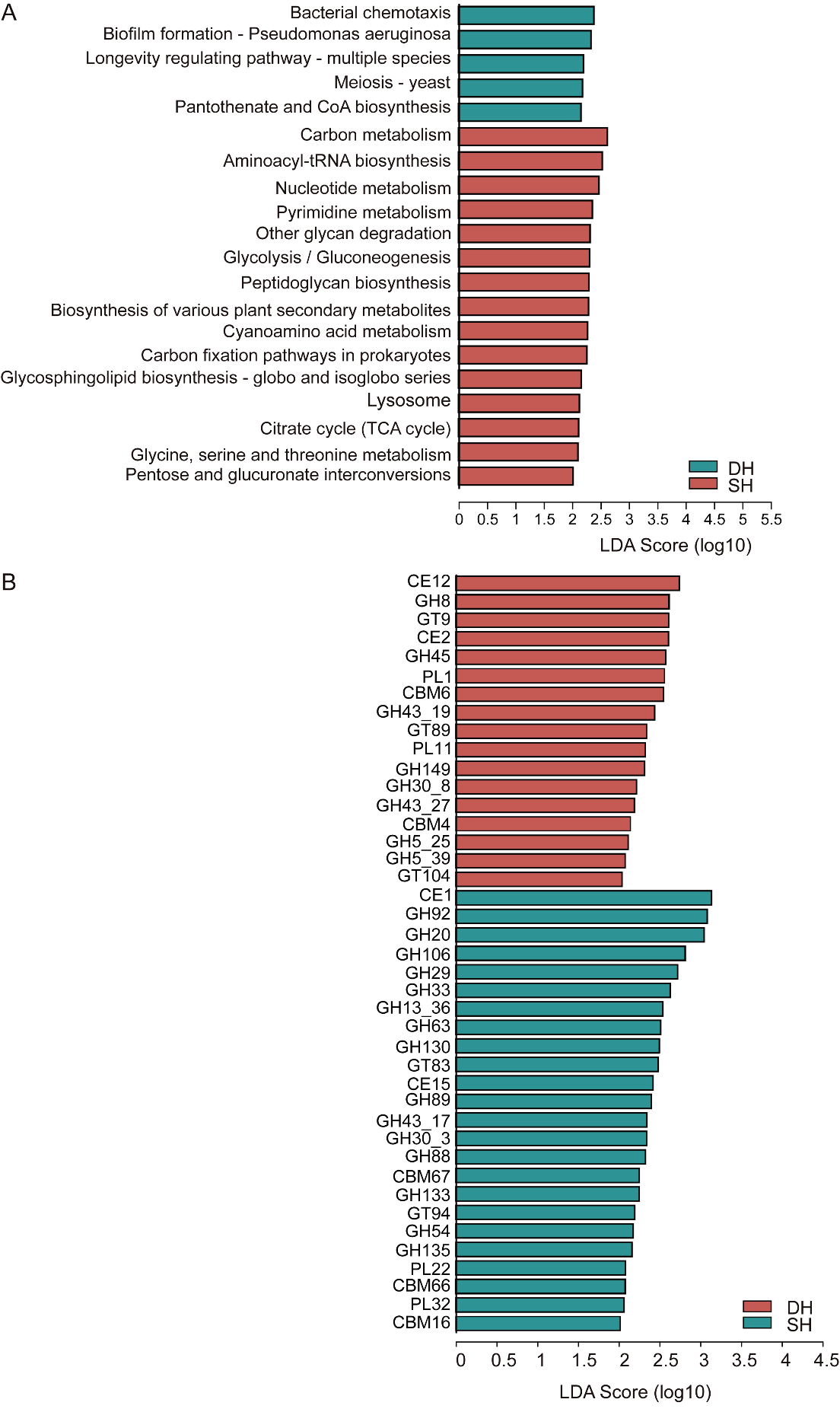


**Supplementary Figure 5** Differential rumen microbiome functions between the DH and SH groups. (A) KEGG functions at the third level. (B) CAZy functions at the family level.


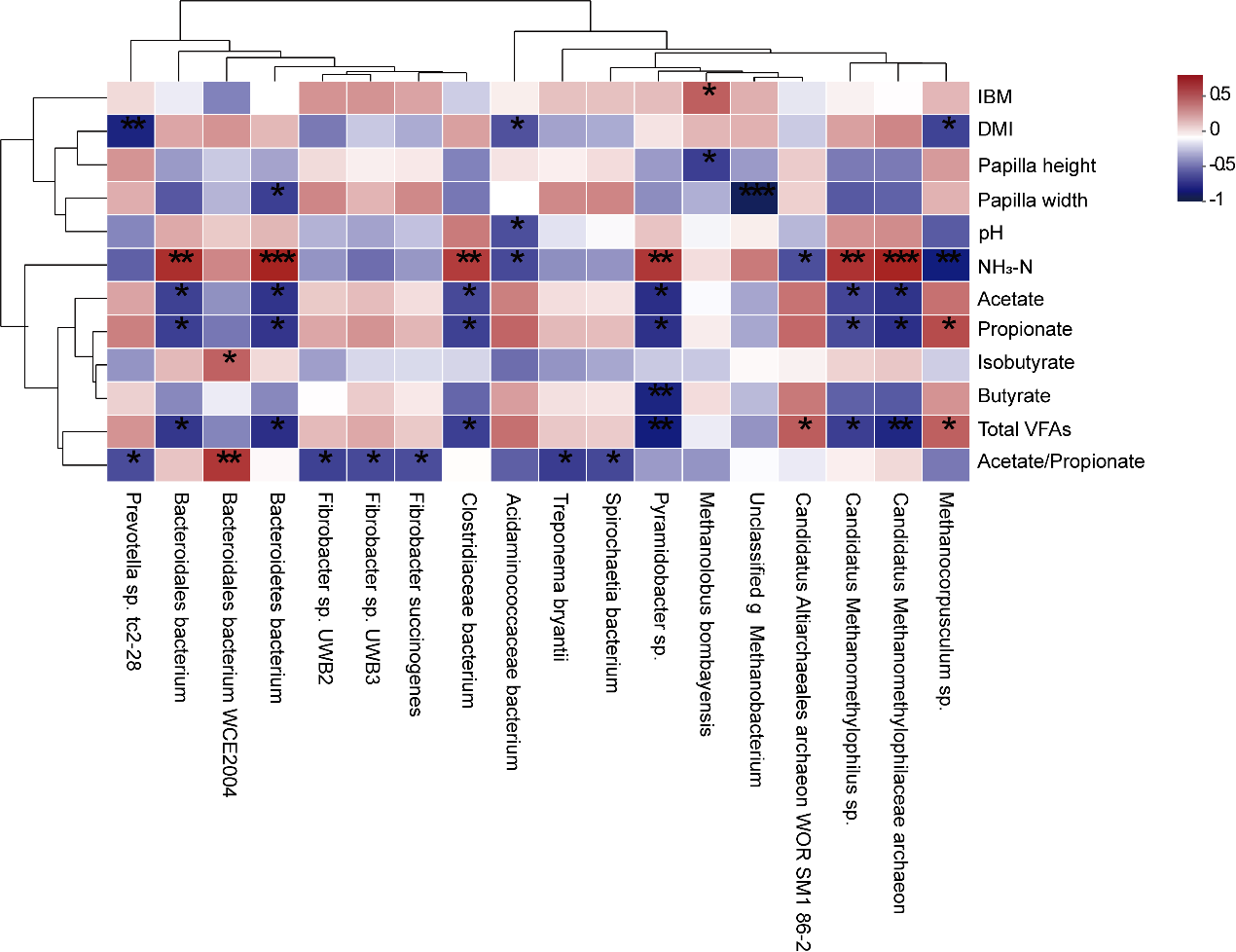


**Supplementary Figure 6** Correlation analysis between growth performance, rumen epithelial parameters, fermentation parameters, and significantly different microbiomes between the DH and SH groups. (|r| > 0.7). n = 7 individuals/group. * *P* < 0.05; ** *P* < 0.01; *** *P* < 0.001.


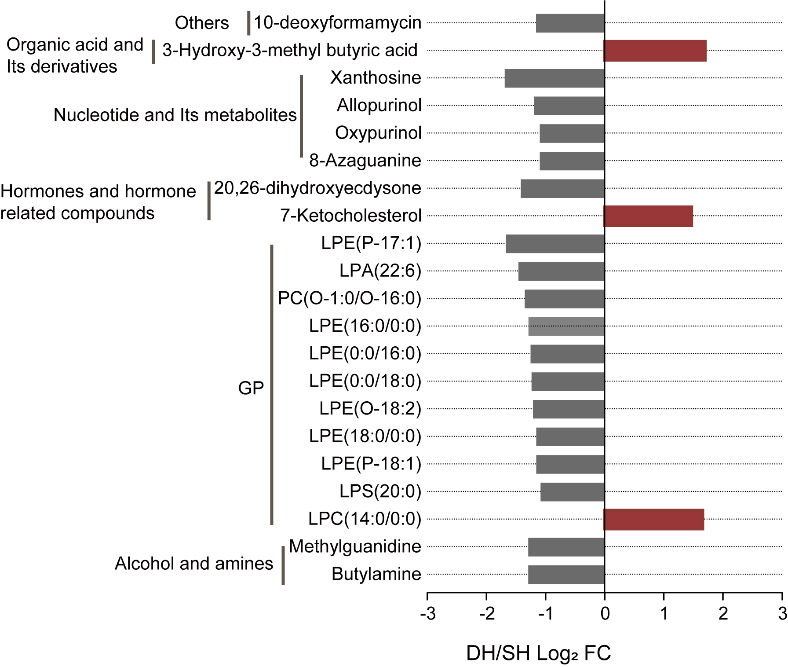


**Supplementary Figure 7** Significantly differential metabolites between the DH and SH groups. n = 7 individuals/group.

**
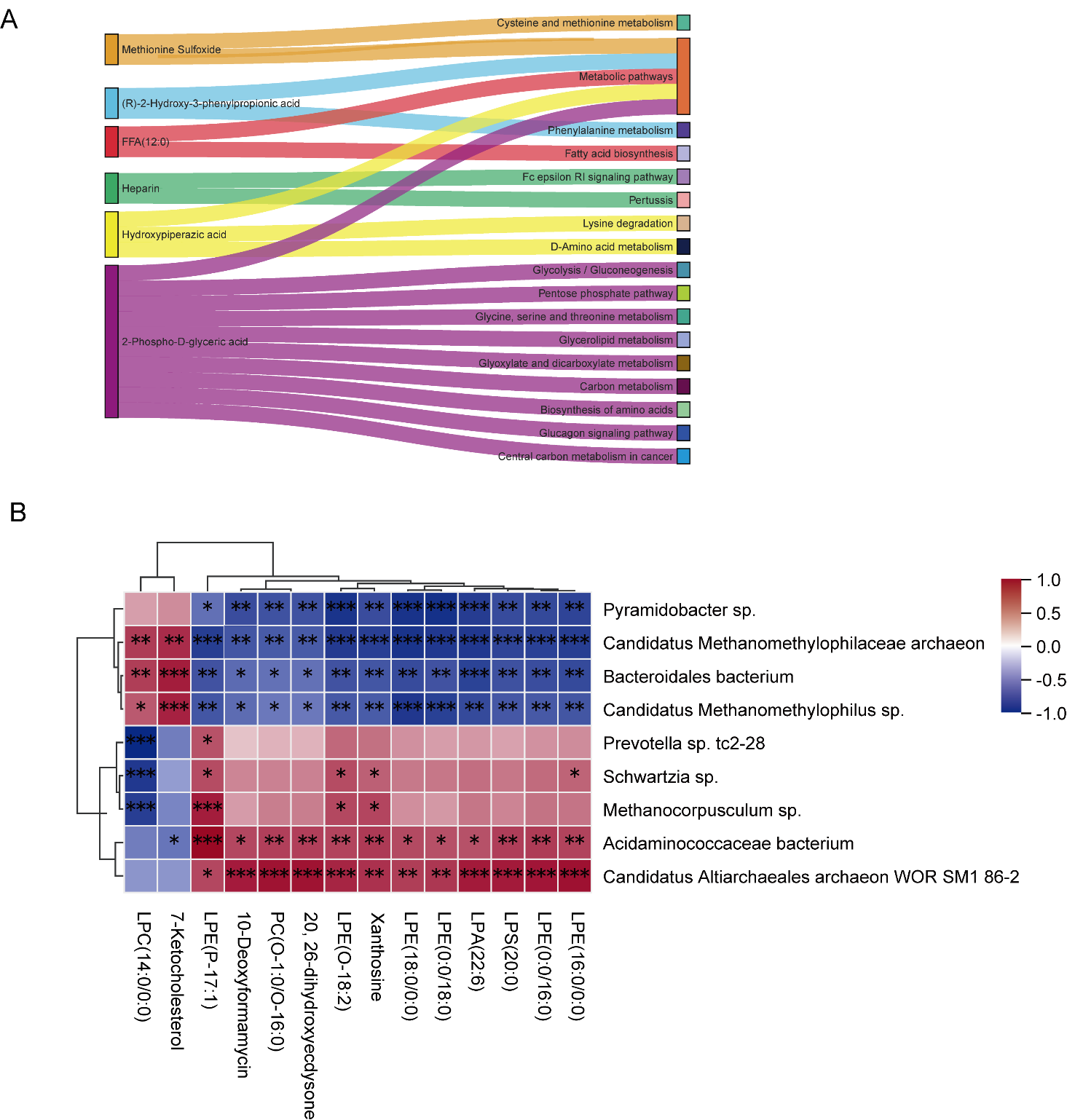
**

**Supplementary Figure 8** (A) Metabolic pathway enrichment analysis between the DH and SH groups. (B) Correlation analysis between microbiome and metabolite profiles in rumen samples between the DH and SH groups. (|r| > 0.8); n = 7 individuals/group.

* *P* < 0.05; ** *P* < 0.01; *** *P* <0.001.
